# Universalized and robust length separation of carbon and boron nitride nanotubes with improved polymer depletion-based fractionation†

**DOI:** 10.1039/d4ra01883d

**Published:** 2024-08-28

**Authors:** Pavel Shapturenka, Benjamin K. Barnes, Elisabeth Mansfield, Matthew M. Noor, Jeffrey A. Fagan

**Affiliations:** a Materials Science and Engineering Division, National Institute of Standards and Technology Gaithersburg MD 20899 USA jeffrey.fagan@nist.gov; b Applied Chemicals and Materials Division, National Institute of Standards and Technology Boulder CO 80305 USA; c Department of Mechanical Engineering and Energy Processes, Southern Illinois University Carbondale IL 62901 USA

## Abstract

Partitioning nanoparticles by shape and dimension is paramount for advancing nanomaterial standardization, fundamental colloidal investigations, and technologies such as biosensing and digital electronics. Length-separation methods for single-walled carbon nanotubes (SWCNTs) have historically incurred trade-offs in precision and mass throughput, and boron nitride nanotubes (BNNTs) are a rapidly emerging material analogue. We extend and detail a polymer precipitation-based method to fractionate populations of either nanotube type at significant mass scale for four distinct nanotube sources of increasing average diameter (0.7 nm to >2 nm). Such separations result in a supernant phase containing shorter nanotubes and a pellet phase containing the longer nanotubes, with the threshold length for depletion decreasing with increasing polymer concentration. Cross-comparison through analytical ultracentrifugation, spectroscopy, and microscopy *versus* applied polymer concentration show tailorable and precise length fractionation for 100 nm through >1 μm rod lengths, with fractionation also designable to remove non-nanotube impurities. The threshold length of depletion is further found to increase for decreasing nanotube diameter at fixed polymer concentration, a finding consistent with scaling attributable to nanotube radial excluded volume. The capabilities demonstrated herein promise to significantly advance nanotube implementation within the scientific community.

## Abstract 2

An improved polymer depletion-based method for length separation enables robust fractionation of boron nitride and carbon nanotubes with consistent scaling.

## Graphical abstract

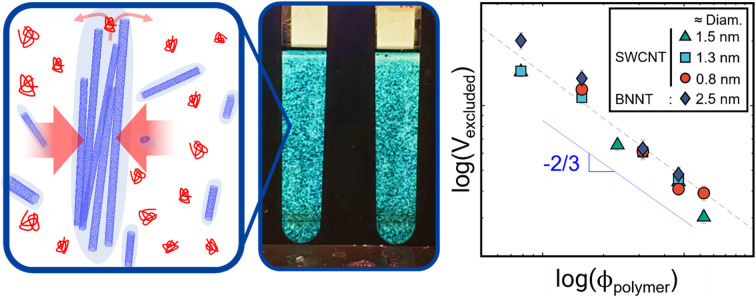

## Introduction

Single-wall carbon nanotubes (SWCNTs) and boron nitride nanotubes (BNNTs) are exemplar nanomaterials with exceptional properties. Many of these properties, such as the electronic band structure and consequent optical spectra, are primarily determined by the diameter of the nanotube, and the orientation of the hexagonal atomic lattice relative to the primary axis typically described by (*n*, *m*) index. However, many others including the functional mechanical, transport, and colloidal properties for applications are governed by the aspect ratio or absolute length.^1–8^ For example, long carbon nanotubes have greater luminescence efficiency and adsorption sites for chemical sensing,^9–11^ offer improved conductivity and structural alignment in high-density thin film architectures for, *e.g.*, transistors,^12–17^ and both SWCNTs and BNNTs yield greater tensile strength when spun into fibers.^18–20^ Short nanotubes, in contrast, are preferred for applications such as bioimaging,^21–23^ in which the nanotubes must be ingested into cells, typically *via* endocytosis, to act as fluorescent reporters.

Despite the promising properties of both SWCNTs and BNNTs for applications, synthesis methods are still developing for both materials. This results in any commercially obtained sample consisting of a polydisperse population containing significant variation in lattice structure, diameter, and length. Solution phase processing to select populations of refined or monodisperse properties is thus of interest and has received significant attention.

Of the two materials, separation technologies for SWCNTs are significantly advanced. In particular, fractionation by diameter, electronic nature, (*n*, *m*) index and even enantiomeric handedness are well established through methods such as aqueous two-phase extraction (ATPE), density gradient ultracentrifugation, and gel chromatography among others.^24–28^ Similar methods are also actively being explored for BNNTs.^29^

**Fig. 1:**
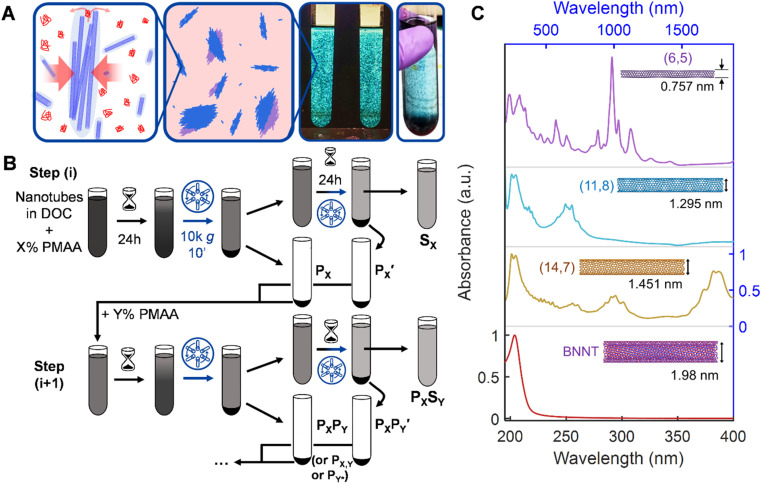
Overview of nanotube length-separation methodology and materials under study. (A) Schematic representation of polymer-based depletion length sorting (PDLS) wherein longer nanotubes form polymer–tube complexes driven by osmotic pressure differences relative to the bulk solution, exchanging their orientational entropy with the translational entropy of the surrounding polymer molecules (small red coils), and readily precipitate. Short nanotubes exclude substantially less volume and therefore remain in solution. The right two photographs demonstrate this complexation and precipitation effect in a highly purified metallic single-walled carbon nanotube (SWCNT) dispersion. (B) Schematic of the PDLS procedure, conducted in the “reverse sequential” precipitation mode (*i.e.*, stepwise decreasing PMAA concentration). Incubation in the presence of polymer induces formation of associated nanotube clusters (tactoids) comprised of longer nanotubes that are separated from short, individually dispersed nanotubes remaining in the surfactant solution *via* a brief centrifugation step. Successive cycles on the supernatant or resuspended precipitate phase at different polymer concentrations allow further length fractionation. (C) Absorbance spectra of all parent nanotube dispersions prior to PDLS. Inset diagrams show representative nanotube species, structures, and diameters for each population. Each spectrum displays sharp, strong absorbance peak structures related to excitonic optical processes and minimal broad wavelength “baseline” absorbance, demonstrating that the nanotube populations are highly purified with few remaining impurities.

Nanotube length separation methods, in contrast, are less extensively researched and the produced methods to date have more restrictions in throughput, selectivity, and precision. The most commonly utilized method for obtaining some control over the length distribution is likely purposeful fragmentation by extended ultrasonication beyond that necessary to individualize nanotubes in an aqueous media; however, this only allows collection of only a short nanotube population, similar to chemical cutting.^23^ Significantly explored methods for length fractionation include size-exclusion chromatography (SEC) and gel chromatography,^30–33^ ultracentrifugation,^9,34^ cross-flow filtration,^35^ acid-assisted condensation,^36,37^ and length-selective polymer depletion-based precipitation. This last technique, also known as “molecular crowding” in the biological sciences, has been demonstrated as a simple length sorting technique in both DNA-wrapped and surfactant-stabilized dispersions (Fig. 1);^38,39^ its improved demonstration and universalization is the focus of this contribution. We believe that the length-selective depletion of nanotube populations by the presence of a low molecular mass polymer, which we term as polymer depletion-based length separation (PDLS), is critically underutilized, with significant advantages in scaling, cost, robustness, and tunability (*vide infra*) over other methods. Likely reasons for this (that we remedy herein) are limited prior demonstrations for only larger SWCNT diameters, no prior demonstration in the most common dispersant–diameter combinations, and no prior demonstration for nanotube materials more broadly, *i.e.*, BNNTs.

The technique itself is entropically-driven; the addition of a small amount of a low molecular mass polymer drives reversible association of nanotubes into fractionable clusters in a strongly nanotube length- and polymer concentration-dependent manner. Separation of these clusters from non-associated nanotubes, *i.e.*, *via* centrifugation, generates macroscopic fractionation into supernatant and pellet phases, containing respectively shorter and longer length nanotube distributions than the parent dispersion. Theoretically, an unlimited number of separations can be conducted to yield finely resolved fractions on arbitrarily large dispersion volumes with high mass throughput. Prior research has established the effectiveness of these separations, and of process choices such as “forward” (increasing polymer concentration in successive fractionations) or “reverse” precipitation strategies (Fig. 1B).^38,39^ While the width of PDLS-separated distributions generally exceeds that of the most resolving methods, (*i.e.*, SEC), the actual limits of multi-stage PDLS have not been explored.

Motivated by the promise of the PDLS method for large scale separations, the missing practical demonstrations on commonly used materials, and gaps in understanding such as a practical scaling between polymer concentration and the depleted rod length threshold, in this contribution we apply and extend the reverse sequential precipitation scheme of PDLS to nanotubes of various diameters and compositions with an advanced multi-stage process. Each of the resulting sets of length fractions are then characterized for their length distributions by imaging and analytical ultracentrifugation (AUC) sedimentation-based methods^40^ to conclusively describe the accomplished separations. Finally, we present and discuss results of experimental exploration and variations to address the limitations of PDLS, and comparison to other methods, providing practical guidelines to the community. Unless otherwise noted, uncertainties are represented by error bars equal to one standard deviation of the reported value.

## Results and discussion

To demonstrate and explore PDLS of nanotube dispersions, we choose nanotube populations from four distinct synthesis methods and two chemistries to cover a broad range of applications. These are: electric arc-discharge (EA) SWCNTs, high-purity ATPE-sorted metallic floating catalyst vapor deposition (FCVD) SWCNTs, smaller-diameter cobalt-molybdenum catalyst (CoMoCAT/SG) synthesized SWCNTs, and BNNTs. This set of materials covers lattice structure diameters from <0.8 nm to >3 nm, probing the extent and effect of depletion forces on length partitioning; we also concurrently probe the effects of different starting nanotube material purity to evaluate process versatility and demonstrate the broad applicability of PDLS. Each source population is both distinct in its average diameter, and in its degree of pre-purification. An electron microscopy (EM) image of the parent material for each population before dispersion is shown in the ESI (Fig. S2†).

In brief, the BNNT nanotube parent was subjected to only a gross impurity and aggregate removal, while the largest (EA SWCNT) and smallest (SG SWCNT) average diameter SWCNTs were rate-zonal ultracentrifugation (RZU) purified to retain only un-damaged and individualized nanotubes;^41^ the intermediate SWCNT diameter population (FCVD SWCNT) was additionally purified to contain only near purely metallic SWCNTs of a narrow (*n*, *m*) species distribution. Details of all sample preparation are reported in the Methods section.

The spectrum of material refinement can be appreciated from the optical absorbance spectra, which are depicted in order of increasing nanotube population diameter in Fig. 1C (see Fig. S1† for absorbance spectra of prior stages of material refinement). Insets feature the (*n*, *m*) species structure that best qualitatively represents the dispersed nanotube population and its approximate average diameter. Each population exhibits sharp and well-defined absorbance peaks related, for the SWCNTs, to the multiple *E*_11_ excitonic optical transitions of semiconducting and metallic nanotubes. In the SG SWCNTs the especially pronounced peaks at 985 nm and 562 nm are attributable to the (6, 5) species, however the sample also contains a variety of semiconducting and metallic species. For the larger diameter SWCNT populations the sharpness of the transitions is increased compared to most literature results due to alkane-filling (Methods) of the inner cores of the FCVD and EA SWCNTs.^42^ The C_7_H_16_-filled EA SWCNTs are the largest of the SWCNT samples in average diameter used in this contribution and are typically distributed in an ≈1 : 2 ratio between metallic and semiconducting species, respectively, with the dominant first order optical (*E*_11_) transitions of the semiconducting species grouped around 1750 nm. The ATPE-sorted, C_24_H_50_-filled FCVD parent dispersion is the most purified of the group, containing only 3 to 4 distinct species in the (1.27 to 1.32) nm diameter range and is estimated to be ≈98% metallic in character. Finally, despite undergoing the least refinement, the BNNT parent dispersion exhibits a single absorbance peak at approximately 204 nm, remaining transparent at visible and near-infrared wavelengths due to the intrinsic wide bandgap of BNNT materials.

To perform length fractionation *via* PDLS we modify the reverse sequential precipitation strategy of Gui *et al.* to overcome limitations in its utility for common use.^39^ These modifications include application of a longer incubation time after each polymer application and addition of a second re-precipitation, “recycling,” step to the processing of each parent supernatant to maximize nanotube mass propagation. Together these modifications significantly increase the overall separation yield, especially for a multistage process. The modified procedure is schematically depicted in Fig. 1B. Briefly, a mixture of weak polyelectrolyte (poly[methacrylic acid], PMAA) and surfactant is mixed with a nanotube dispersion in a centrifuge tube to reach a given specific concentration, *X*%, of PMAA expected to differentially deplete longer from shorter nanotubes in that dispersion. Specific details of the applied PMAA concentrations are tabulated throughout the text and reported in the ESI (Table S1†); a surfactant concentration of 10.0 g L^−1^ sodium deoxycholate (DOC) is held constant for all dispersions. These dispersions are then incubated at ambient laboratory temperature (≈22 °C) for 24 h before centrifugation at 1047 rad s^−1^ (or ≈10 000*g*, in which *g* = 9.81 m s^−2^) except the SG SWCNTs, which were spun at 1309 rad s^−1^ (or ≈15 000 *g*), for 10 min. The resulting supernatant is transferred (decanted) to another centrifuge tube to sit quiescently for an additional 24 h before re-centrifugation and separation. The precipitate phase from both the first centrifugation (*P*_*X*_) and second centrifugation 
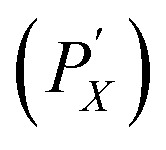
 are re-dispersed with a small amount of fresh 10 g L^−1^ DOC solution and combined. In a multistage “reverse” cascade, the re-dispersed pellet is mixed with polymer solution to concentration *Y*%, becoming the new “parent,” while the supernatant (*S*_*X*_) is reserved as a finished fraction (or can be further fractionated if desired, *vide infra*). In a multistage process we append the descriptors of subsequent stages, *e.g.*, *P*_*X*_*S*_*Y*_ and *P*_*X*_*P*_*Y*_, where the latter can be shortened to *P*_*X*,*Y*_, or, for further brevity, *P*_Y*_ (used herein), to reference only the most recent polymer concentration the dispersion was precipitated by. In this work we demonstrate cascades comprised of four total stages, applying decreasing PMAA concentration at each successive stage. This yielded five fractions, wherein the final precipitate is simply resuspended in aqueous DOC solution.

Demonstration that the separation of the pellet and the supernatant phases in this process is due to precipitation and not sedimentation of individual SWCNTs is provided in Fig. S3.† In this figure, cross-polarized microscopy images of surfactant-stabilized SWCNTs interacting with 5 kDa PMAA (*R*_g_ expected to be ≈1.5 nm)^43^ in thin, sealed fluid volumes directly show formation of domains with significant nematic character (tactoids). These domains are what are separated by the low-speed centrifugation. In addition, the observed condensation behavior conclusively shows that SWCNTs yield substantial amounts of their hydrodynamically occupied, or scribed, dilute-phase volume to the polymer. In contrast to classical Asakura–Oosawa theory with colloidal spheres,^44,45^ this implies that nanotubes will be depleted across an expanded window of polymer concentration, enabling tailorable size resolution.

**Fig. 2:**
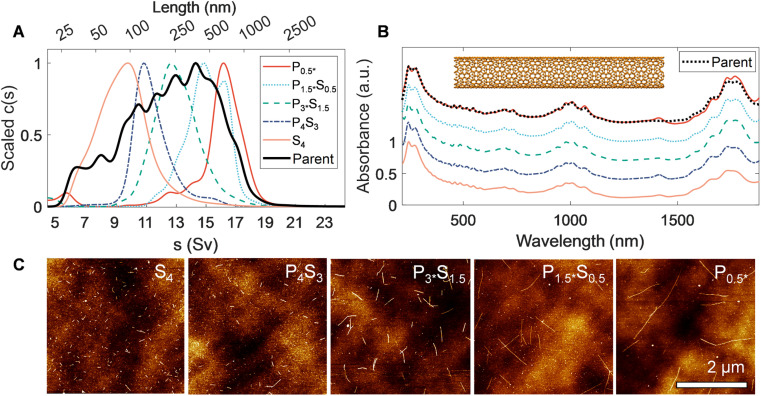
Characterization of canonical rod-like SWCNTs separated *via* PDLS. Rate zonal ultracentrifugation (RZU) refined, heptane-filled, EA SWCNT fractions are compared to the parent distribution. (A) Sedimentation coefficient distributions obtained from fitting absorbance-based AUC time-dependent radial concentration profiles. The peak of each mass-weighted distribution observed by AUC for the sorted population and parent sample are scaled to ease comparison of the relative shifts between populations. These shifts demonstrate dramatic partitioning and population narrowing, particularly visible on the non-linearly scaled length (top) axis. (B) Scaled ultraviolet, visible, and near infrared (UV-vis-NIR) absorbance spectra of the parent dispersion and separated fractions, showing a weak, but discernible, trend of increasingly prominent optical transitions with increasing nanotube length. The spectra are scaled to one at *λ* = 247 nm and are offset by 0.3 between fractions, except for the *P*_0.5*_ fraction, which is shown at the same offset as the parent dispersion. (C) Representative atomic force micrographs of resulting EA SWCNT length fractions.

A key step to demonstrate fractionation is measurement of the resulting nanotube length distributions. Notably, measurement of dispersed properties such as size (*i.e.*, nanotube length and/or diameter) is still technically challenging for nanomaterials, even with refined populations. The measurements herein are even more challenging; the different average diameters of the nanotube parents utilized affect their characterization by modulating factors including their observability in microscopy, their mechanical stiffness, and their hydrodynamic transport properties.^46,47^ The EA nanotubes (〈*d〉* ≈ 1.45 nm) are the simplest nanotube system to characterize of those in this study due to their larger diameter and stiff rod morphology; (persistence length >100 μm). The hydrodynamic behavior has also previously been characterized by AUC and atomic force microscopy (AFM) and cross-correlated.^40,46^ The EA SWCNTs are thus a prime candidate for benchmark comparisons. Fig. 2 shows AUC sedimentation coefficient distributions, absorbance spectra, and AFM micrographs measured from the PDLS length-separated EA nanotube populations. Representative electron microscopy images of separated fractions, the preparation of which does not readily allow for length determination of individual nanotubes in the length range of interest to this contribution, are shown in the ESI (Fig. S13†).

In AUC, the sedimentation coefficient of a dispersed nanotube monotonically increases with its length, with quantitative conversion performed using rod hydrodynamic theory and separately experimentally determined particle density (*ρ*) and radial hydrodynamic shear plane (*R*_h_) values (see Table 1 for details).^40^ The value of AUC is that it measures the sedimentation coefficient and length distributions from a macroscopic ensemble contributed to by trillions of particles even in dilute measurement. The observed range also covers the entire span of SWCNT lengths in a single measurement, making for easy and direct intercomparison. For the EA SWCNTs in Fig. 2, the (absorbance-measured and thus mass-weighted) length distribution of the initial parent dispersion is broad, as expected for an ultrasonication dispersed sample, with a mean length of 330 nm and a substantial length polydispersity index (LPDI) of 1.79 (LPDI calculated analogously to a macromolecular polydispersity index, *i.e.*, as a ratio of mass- and number-averaged lengths).

**Table 1:** Relevant referenced and determined physical parameters of the various SWCNT populations in 10.0 g L^−1^ DOC at 20.0 °C used for AUC data fits and length calculations

	*ρ*_anhydrous_ (kg m^−3^)	*ρ*_buoyant_ (kg m^−3^)	*ρ*_solution_ (kg m^−3^)	*μ*_solution_ (mPa s)	*R*_h_ (nm)
C_7_H_16_@EA SWCNTs^16,40^	1450	1140	1000.42	1.056	3.5
C_24_H_50_@FCVD SWCNTs	1490	1092			3.05
H_2_O@SG SWCNTs^16,48,49^	1550	1067			2.7

Separated fractions, in contrast, display dramatically narrowed distributions and the expected trend of increasing sedimentation coefficient, and thus nanotube length, with each added PDLS stage. Even for a simple 5 step cascade the fractions span nearly an order of magnitude in separated average length. Notably, since the initial mass was distributed across a broad length range in each nanotube population, the longest and shortest separated fractions demonstrate particularly large enrichment factors.

We report the quantified average number-weighted nanotube length from each fraction in Table 2. The average length of the first fraction in our reverse-direction cascade (*S*_4_) measured 108 nm, and the final precipitated population (*P*_0.5*_) at 620 nm. Because it was the longest fraction, however, many nanotubes exceeded 1 μm in length, and further separation could have been conducted to resolve an even longer average length population. Length distributions determined by AFM (representative AFM images in Fig. S9 to S12†) and AUC were in reasonable agreement, with average values from both methods falling within a 50 nm range for most fractions. The LPDI values for all the fractionated distributions, measured by either AFM or AUC, averaged values of ≈1.2. For both techniques, the measurement uncertainty is believed to be small relative to the distribution widths. In AFM uncertainties can be bounded by several pixels ([10 to 40] nm, depending on image resolution), while in AUC uncertainties convolve discretization error and uncertainty in the *s*-value to length conversion. The uncertainty in AUC derived values will thus vary across the absolute length range but is anticipated to be of <10 nm scale for most of the length range. Because of their magnitude relative to the distribution width, these uncertainties are not propagated into tabulated length values.

**Table 2:** Average number- and mass-weighted lengths (each averaged from AFM and AUC measurements), upper/lower quartile values (denoted with “75%” and “25%” subscripts, respectively), and length polydispersity indices (LPDIs) for successive polymer-precipitated fractions of arc-discharge SWCNTs. Length values were cross-validated using data from large-sample AFM contour length measurements and AUC fitting. Notably, the absolute length and polydispersity values obtained with both methods are in excellent agreement, underlining the suitability of EA SWCNTs as a model rodlike nanoparticle system

	[PMAA] (g L^−1^)	〈*L*_*N*_〉 (nm)	*L*_25%,AFM_ (nm)	*L*_75%,AFM_ (nm)	*L*_25%,AUC_ (nm)	*L*_75%,AUC_ (nm)	〈*L*_M_〉 (nm)	LPDI (AFM)	LPDI (AUC)
*P*_0.5*_	—	619.8	435.2	908	599	939	752.2	1.18	1.23
*P*_1.5*_, *S*_0.5_	5	453.7	323.1	664.5	424	707.5	536.8	1.18	1.19
*P*_3*_ *S*_1.5_	15	246.7	188.8	303	225.7	450.6	295.8	1.15	1.25
*P*_4_ *S*_3_	30	164.2	134	204.5	131.3	262.3	194.1	1.11	1.2
*S*_4_	40	107.6	90.5	139.8	81.2	182.8	133.8	1.24	1.27
Parent	—	189.4	—	—	287	751.3	339.6	—	1.79

Optical properties of the length-sorted EA SWCNT fractions (Fig. 2B) provide additional insight into material quality and the possibility of other, non-length, selectivity during PDLS. As frequently observed in prior studies, the *E*_11_ transition strength in the absorbance of the longest nanotube fraction (*P*_0.5*_) is more prominent relative to the general broad wavelength “π-plasmon” absorbance of most nanocarbon than the parent dispersion (that of the shortest, *S*_4_, is suppressed accordingly). For this diameter range of SWCNTs, past contributions attribute the source of this effect mostly to differential fractionation of remaining impurities and defective nanotubes,^31^ although with the possibility of additional weaker length-dependent effects. All fractions also display highly consistent spectral peak distributions with only a slight *E*_11_ band reweighting toward longer wavelengths for the longest tube fractions. The latter is possibly suggestive of mild diameter selectivity over the course of the sorting process, but one not specific enough to strongly affect the fractionation of the SWCNT diameter distribution. Both observations imply limited differential enthalpic interactions between different (*n*, *m*) SWCNTs and PMAA in the depletion process. In agreement, individual SWCNTs imaged by AFM displayed a consistent, tightly distributed set of step heights (Fig. S5†); the lack of damaged nanotubes imaged for any fraction is attributable to the prior rate-zonal purification, which selected for structurally pristine, individualized nanotubes.^31^ Absorbance measurement of the SWCNTs in the presence of polymer, or summed post fractionation, reveal no modification to the peak structure, positions, widths, and intensities, indicative that effects of PMAA addition do not include chemical reactivity.

A significant advance of this contribution is a marked improvement in total mass yield of longer fractions and concomitant reduction in early fraction polydispersity. This is achieved through longer incubation, and inclusion of the re-incubation and precipitation, “recycling” step, in the PDLS process. The addition of the recycling step is shown schematically in Fig. 1B. This step follows observation that singly-decanted supernatants will continued to show additional precipitation if left to sit quiescently. The effect is traceable to mass transport inefficiency in the separation process. Details and comparisons of mass retention and separation yield with and without recycling, as well as the mass recovered with recycled precipitates can be found in Fig. S4 and Table S2 of the ESI.† The inclusion of recycling typically increases mass throughput, sometimes by values up to 20% to 50% depending on functional details of concentration and centrifugation parameters, with concomitant narrowing of resulting, left-behind, supernatant fraction distributions. Such throughput is generally not observed to negatively increase the precipitate polydispersity either; in comparison of the breadth of our *P*_1*_S_0.5_ fraction to a plasma-torch SWCNT fraction from Gui *et al.* (Fig. S6†),^39^ we see equal or narrower distribution width for our longest EA sample.

**Fig. 3:**
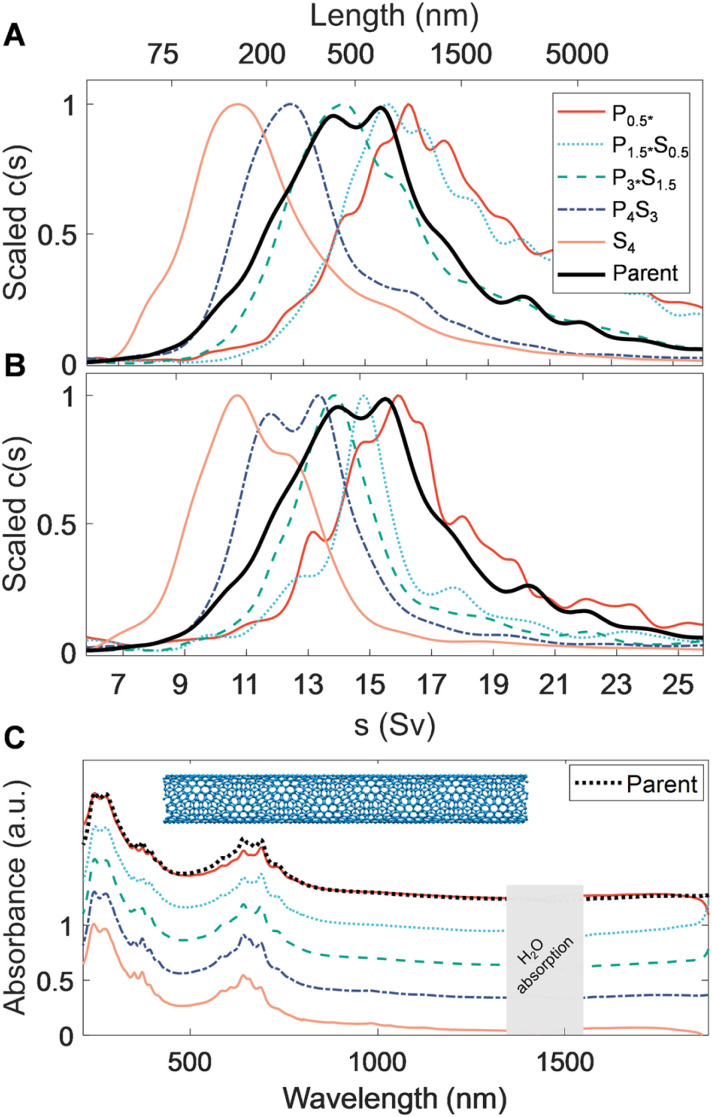
PDLS of highly purified (>98% metallic and species-sorted) tetracosane-filled FCVD SWCNTs. (A) Sedimentation coefficient distributions obtained from absorbance-based, time-dependent, radial concentration profiles *via* AUC, obtained after ultrafiltration (to remove the PMAA depletant) and resuspension in aqueous DOC solution. Similar trends are observed as for the EA SWCNTs, showing distinct, narrow length fractions. (B) AUC runs of the same fractions after 30 s of tip ultrasonication, showing dramatically reduced right tails in all fractions. Ultrasonication likely aided in disassociating bundled and loosely aggregated SWCNTs that were compacted during ultrafiltration. (C) Scaled UV-vis-NIR optical absorbance spectra. Mild reweighing of metallic transitions in the 600 nm to 700 nm region suggests small diameter selectivity trends between fractions. The spectra are scaled to one at *λ* = 247 nm and are offset by 0.3 between fractions, except for the *P*_0.5*_ fraction, which is shown at the same offset as the parent dispersion.

We now extend PDLS separations to previously unstudied SWCNT populations, explicitly one previously metallicity- and species-separated, and a second small-diameter population. To represent species separated SWCNTs we utilize an intermediate diameter FCVD SWCNT (〈*d*〉 = 1.25 nm) sample purified for metallicity and reduced (*n*, *m*) species number by ATPE. A key feature of this material is that its smaller diameter leads to a diminished persistence length relative to the EA SWCNTs (*L*_P_ ≈ (50–100) μm), although they can still be regarded as mechanically stiff. Sedimentation and optical extinction properties of the fractionated FCVD populations are shown in Fig. 3. The average mass-weighted lengths of the initial and final (mass-abundant) separation stages were approximately 239 nm and 678 nm (full length details in Table 3). Mass propagation into the final *P*_0.5*_ precipitate was minuscule, presumably due to a lack of longer nanotubes in the parent population. However, AUC still measures an average length of 780 nm for this fraction, demonstrating that selective fractionation of long SWCNTs by the PDLS method is continuing. Overall, the separated fractions are well spaced in length, and display reasonably sharp left-tail (short) cutoffs in the fit sedimentation coefficient distributions. AFM-measured LPDI values also appear to be relatively constant, suggesting improved length homogeneity relative to the parent.

**Table 3:** Average length values for PDLS sorted FCVD and SG SWCNT as well as BNNT populations. Subscripts denoted “25%” and “75%” indicate lower and upper quartile length values, respectively. The elevated AUC-derived LPDI values for FCVD SWCNTs relative to those obtained with AFM are believed to originate from limitations of the AUC method, namely (i) hydrodynamic interactions of long SWCNTs impeding ideal, independent sedimentation, and (ii) residual small aggregates obtained during the ultrafiltration process after the conclusion of PDLS. Despite the presence of a broad diameter range leading to additional complexity in the correlation between depleted BNNT length and polymer concentration, robust BNNT length fractionation and polydispersity reduction is observed in AFM-determined values

Nanotube type	Fraction	[PMAA] (g L^−1^)	〈*L*_*N*_〉 (nm)	*L*_25%,AFM_ (nm)	*L*_75%,AFM_ (nm)	*L*_25%,AUC_ (nm)	*L*_75%,AUC_ (nm)	〈*L*_M_〉 (nm)	LPDI (AFM)	LPDI (AUC)
FCVD	*P*_0.5*_	—	443.4	—	—	472.6	2323.4	779.2	—	1.76
	*P*_1*_ *S*_0.5_	5	489	417.6	768.5	378.2	1309.6	677.6	1.21	1.68
	*P*_2*_ *S*_1_	10	407.7	307.5	579.2	283.9	838.5	544.4	1.25	1.44
	*P*_3_ *S*_2_	20	270.65	199.2	323.1	176.1	443.5	375.1	1.31	1.48
	*S*_3_	30	175.0	151.6	242.6	111.8	277.2	238.6	1.18	1.62
	Parent	—	338.6	—	—	302.6	1190.3	540.7	—	1.60
SG	*P*_1*_	—	820	376.4	800.2	744.6	1724.4	992.7	1.2	1.15
	*P*_2*_ *S*_1_	10	604.8	395.5	713.6	509.6	1315.2	746.1	1.24	1.23
	*P*_3*_ *S*_2_	20	372.3	233.9	366.9	330.4	1003.1	495.9	1.25	1.4
	*P*_4_ *S*_3_	30	265.5	144.7	245.3	214.2	924.8	388.2	1.17	1.63
	*S*_4_	40	201.8	118.8	234.8	146.6	599.5	333.6	1.36	1.92
	Parent	—	512.3	—	—	509.6	1974.6	791.7	—	1.54
BNNT	*P*_0.5*_	—	500.9	220.8	614.6	—	—	716	1.36	—
	*P*_1*_ *S*_0.5_	5	622.2	463.2	722.5	—	—	719.8	1.26	—
	*P*_2*_ *S*_1_	10	409.4	333.8	481.1	—	—	453.7	1.1	—
	*P*_3_ *S*_2_	20	172.5	136.9	226.4	—	—	203.4	1.18	—
	*S*_3_	30	137.4	93.6	172	—	—	169.9	1.21	—
	Parent	—	397.7	266.0	498.0	—	—	502.4	1.26	—

Comparison of the ultraviolet, visible, and near infrared (UV–vis–NIR) absorbance spectra among the separated fractions (Fig. 3C) show that the peaks in the *M*_11_ transition region (550 nm to 750 nm) of each length-sorted fraction appear to be sharper than that of the parent dispersion. This indicates a more consistent surfactant-mediated dielectric environment and possible amplification of a given species through diameter or chiral selectivity. Indeed, a gradual reweighing of the dominant *M*_11_ transition from a single, dominant peak at 640 nm to a doublet at 630 nm and 690 nm is observed, further supporting species selectivity and reducing the relative intensity of any single peak when scaled to the parent spectrum. It is unknown if the source of these observations is a selective enthalpic interaction with the PMAA, or comes from differential (*n*, *m*) species length distributions in the parent dispersion. Owing to the narrow range of prominent *M*_11_ transitions in the visible range for the parent dispersion, all FCVD SWCNT dispersions appeared a saturated cerulean color (see Fig. S7†). This narrowness of the absorption enables direct visualization of the structures formed during the precipitation process at SWCNT concentrations for which the other SWCNT populations are effectively non-transparent through the path length of the utilized centrifugation tubes. These include a variety of discernible discrete and continuous macroscopic structures spanning the entire settling tube, observed after 24 h of each separation stage. The nanotube condensates forming over the course of the first separation stage were particularly remarkable, establishing a single dendritic structure coaxially with the tube.

Interestingly, cross-comparison of sedimentation, spectroscopic, and microscopy characterization data of these highly purified FCVD fractions suggests nontrivial aggregation is present in all the fractions of the FCVD population. However, since fractionation occurred (*vide infra*), this association or aggregation must have occurred after the PDLS processing, most likely during the ultrafiltration-based removal of the residual PDLS polymer for reasons to be discussed. Focusing on the AUC data, when compared to the sedimentation fits of the EA SWCNT fractions in Fig. 2, all FCVD fractions in Fig. 3A exhibit a noticeably more pronounced right tail. To test whether these tails are the result of a bias or decrease in efficiency of the PDLS method AUC measurements were repeated on the same samples after applying 30 s of additional tip ultrasonication to re-individualize aggregated structures (<1/60th the initial energy input of dispersion). The results of this experiment, presented in Fig. 3B, is conclusive. The right-side tails are markedly reduced to almost eliminated across all fractions without any increases to the distribution ranges in which short (*i.e.*, newly broken) nanotubes would be observed. Atypically for DOC dispersed SWCNT samples, resuspension and remeasurement *via* AUC of the sedimented SWCNTs from the extra-sonication aliquots yields a re-emergence of the right-side tail. Together these results strongly indicate that the issue is one of an aggregative bundling of the separated SWCNTs after the PDLS process, as a concomitant process would likely propagate a significant short nanotube population to later fractions in the reverse schema in a manner not observed.

Supporting these observations are results from AFM. An abnormal nanotube cross-section step height to length correlation was found for a significant portion of AFM-imaged FCVD fractions (Fig. S5†), with tubular objects of 100 nm to 1000 nm in length measured to be multiple nanotube diameters tall. However, the average values and LPDI metrics continue to show distinct fractionation and significant reduction respectively. In comparison the LPDI values from AUC measurements diverge from those of the AFM measurement, especially in the shorter length-sorted fractions, due to false interpretation of faster bundle sedimentation as the presence of much longer nanotubes. As to why this SWCNT population aggregates in post separation processing and the larger EA and smaller SG (*vide infra*) do not, we attribute the effect to the high purity in metallicity of the separated objects, and in a reduction in bound DOC coverage previously identified for this diameter range.^50^ Metallic SWCNTs are expected to experience stronger attractive van der Waals interactions to each other, due to a greater degree of polarizability, than to a semiconducting SWCNT or as between semiconducting SWCNTs, with an additional amplification from the species separation making each tube–tube encounter an interaction between more alike approaching objects.^8^ That this diameter range of nanotubes is known to have a lesser DOC surface packing density, probably lastly reduces the barrier for rearrangements leading to aggregation, particularly compared to the dense surfactant layers observed on smaller diameter SWCNTs.^48–51^

Despite the (readily addressed) additional difficulty the post-PDLS process aggregation adds in processing these species-refined and highly metallicity pure samples, achieving equally well-spaced length fractionation as with less refined populations demonstrates the ultimate versatility and broad implications of PDLS. In such high-value samples, processes that even potentially lead to significant mass loss during separation process are untenable. With PDLS, it is possible to recover all nanotube mass without fear of loss, allowing material pipelines for multiple end uses (*e.g.*, metallic *versus* individual (*n*, *m*) semiconducting species) to originate, and be efficiently and precisely co-(or differentially) processed, from the same initial material lot.

### Length fractionation of narrow diameter SWCNTs

**Fig. 4:**
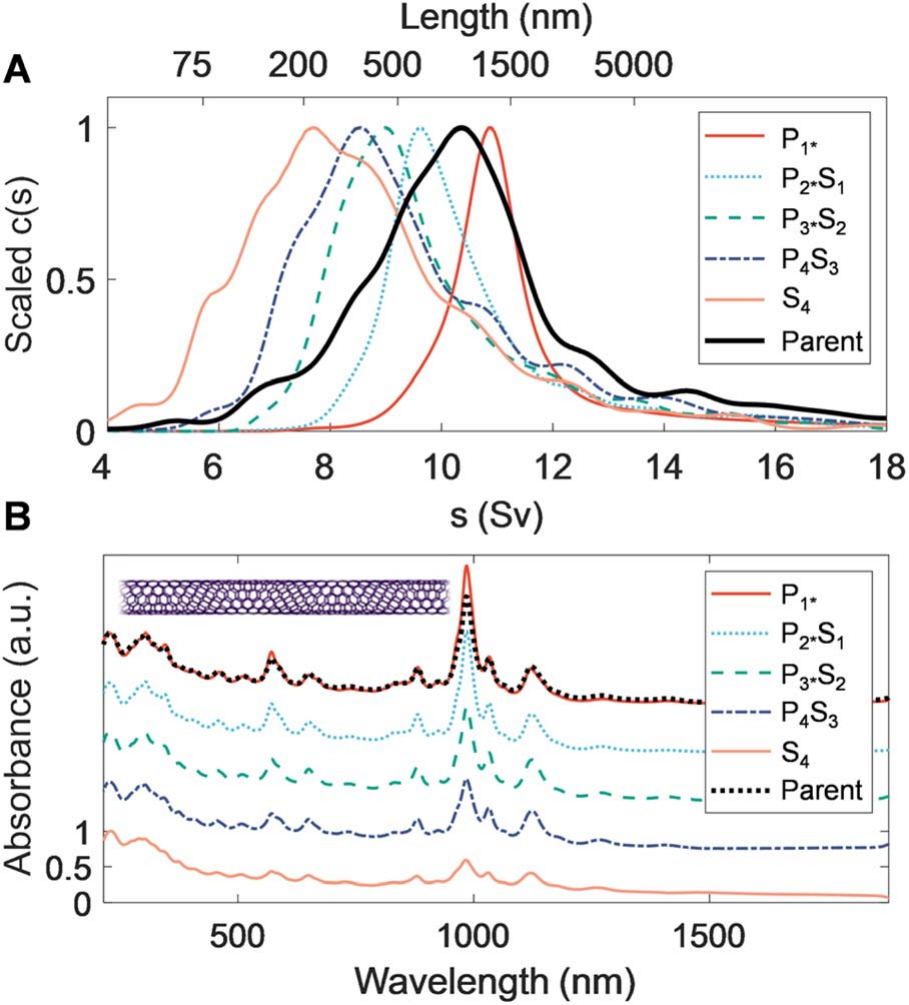
Characterization of separated length fractions of RZU-refined, small-diameter SG SWCNTs. (A) Sedimentation coefficient distributions obtained from time-dependent radial AUC absorbance profiles. (B) Scaled UV-vis-NIR absorbance spectra showing distinct sharpening of the (6, 5) *E*_11_ and other (*n*, *m*) species excitonic transitions with increasing mean length within fractions. The spectra are scaled to equal one at *λ* = 227 nm and are offset by 0.7 between fractions, except for the *P*_1*_ fraction, which is shown at the same offset as the parent dispersion.

Extending PDLS to an even smaller average diameter SWCNT population, with a species distribution strongly biased to the (6, 5) SWCNT, is similarly technologically important. Specific (*n*, *m*) species in this population are the most commonly separated species, and are used for sensing and optoelectronic purposes, both of which have preferred length distributions based on the specific application. Efficient length separation methods are thus particularly valuable for this diameter range. Table 3 shows the average lengths and polydispersity of the fractions resulting from each PDLS separation stage; the 〈*L*_*N*_〉 of the initial (*S*_4_) and final (*P*_1*_) stages are 201 and 820 nm, respectively. This is a significantly elevated length range for the same nominal polymer concentration compared to the larger diameter SWCNTs considered. This increase also coincides with a divergence in length dispersity as measured by AFM and AUC; the sedimentation-derived LPDI monotonically decreases with each successive fraction while the AFM-measured dispersity values stay constant and relatively suppressed. One hypothesis for this divergence is that the interpretation of the faster-sedimenting part of the AUC-derived distribution tail as belonging to well-individualized (very long) straight SWCNTs is incorrect. At this extreme of the SWCNT diameter range (〈*d*〉 ≈ 0.76 nm), SWCNTs are significantly flexible (*L*_p_ ≈ 5 μm to 10 μm). The right-side tail most likely represents sedimentation at rates inconsistent with rigid-rod hydrodynamic theory due to this flexibility. Due to the slope of the rigid-rod hydrodynamic theory, characterization of long-length, small-diameter nanotubes become increasingly imprecise around 800 nm, as a rapid divergence in predicted length for small changes in sedimentation rate occurs. Flexibility increases rod sedimentation by decreasing the experienced average friction coefficient, and as such over-estimation of length for smaller-diameter nanotubes with AUC has been reported for the (6, 5) SWCNT and attributed to the reduced persistence length.^52^ The sedimentation profile of the *P*_1*_ fraction in Fig. 4 separately shows a different, also known, non-ideality effect for very extended rods. This effect arises from the hydrodynamic interactions between very extended rods and is observed in this sample because the narrower diameter SWCNTs have the least mass per unit length; such interactions narrow and shift the sedimentation coefficient distribution to lesser values. This results in under-estimation of the length distribution.^52^ For this measurement the sample was diluted towards the effective signal-to-noise limit of our AUC to reduce the effect, but the sedimentation distribution is still mildly affected.

Optical extinction of the SG population PDLS fractions, shown in Fig. 4B, displays a consistent distribution of peaks reflecting that no significant diameter or species separation occurs along with the length separation. The most prominent feature at 985 nm for each of the length-separated fractions is assignable to the (6, 5) *E*_11_ transition. As with the other nanotube source populations, the *E*_11_ and *E*_22_ modes sharpen and their scaled intensity grows relative to the UV-wavelength sp^2^ carbon/plasmon peak for each successive longer length population; the trend with increasing length is, however, stronger than that of the EA SWCNTs. This is also consistent with observations in prior work and is primarily attributable to a reduced prevalence of defective nanotubes and non-nanotube material that tend to collect in the shorter fractions.^31^

### Length fractionation of BNNTs

Relative to the SWCNT populations described above, BNNTs are larger in diameter and are significantly stiffer nanoparticles. Their as-synthesized populations are typically multiwalled, and exhibit more polydispersity in diameter and wall number than SWCNTs, though synthesis and refinement processes are rapidly advancing.^29,53,54^ Owing to the lack of a selection methodology for primarily individualized and structurally pristine nanotubes (as was performed for SWCNT fractions *via* rate-zonal centrifugation), the BNNT parent dispersion fractionated by PDLS contained a broader diversity of shapes, bundles, damaged nanotubes, and non-nanotube entities present that were expected to influence the length distributions and overall partitioning of mass among the PDLS fractions. Instead, despite this additional polydispersity, we find that BNNT dispersions are readily fractionable into well-refined populations *via* PDLS.

**Fig. 5:**
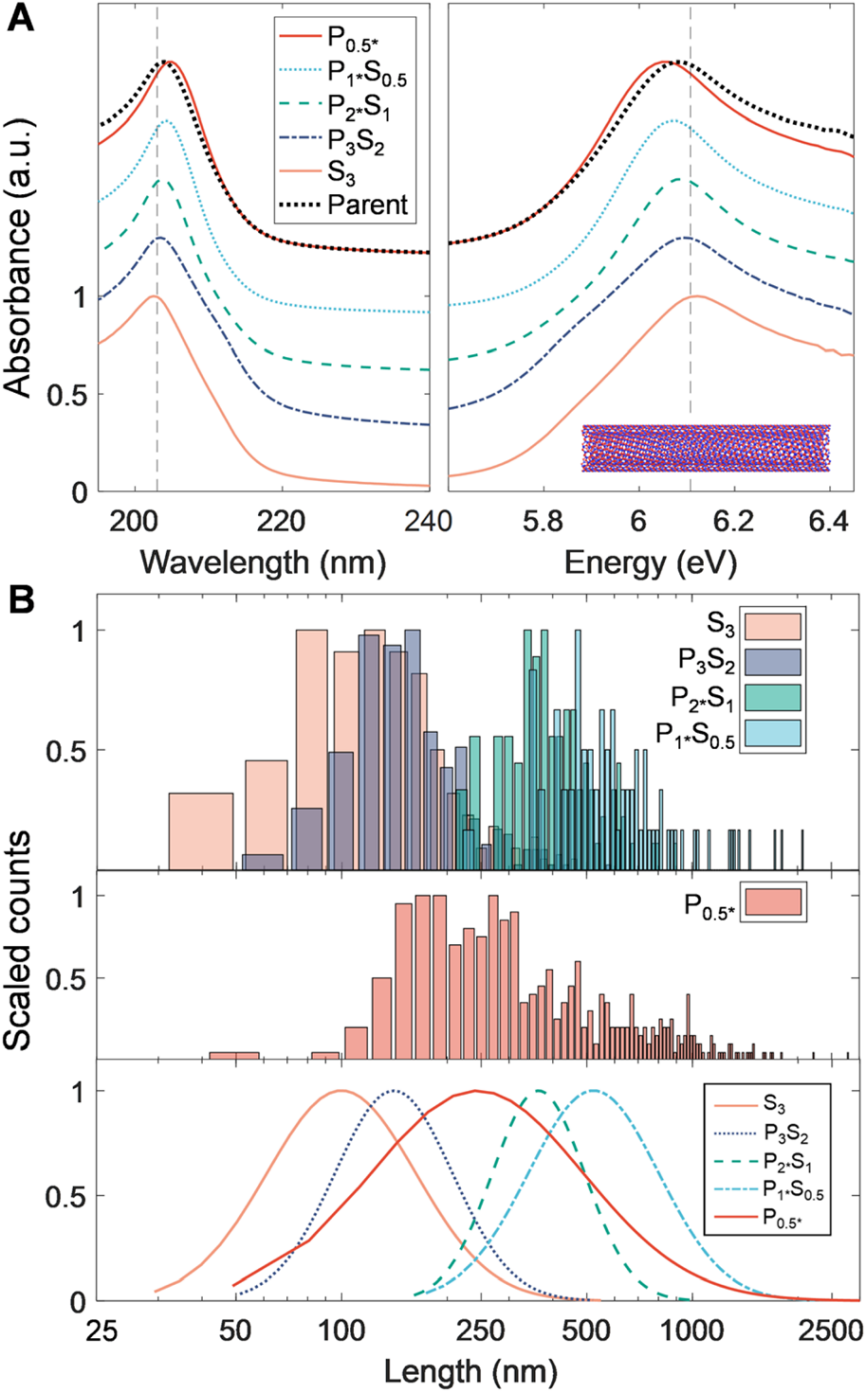
PDLS-separated BNNTs. Nanotube material was obtained from a dispersion decanted from sonicated and centrifuged material. (A) Absorbance spectra (plotted *versus* wavelength and energy) of all separation fractions, scaled to unity at their primary UV absorbance peak (approximately 204 nm), and offset by 0.3 between fractions, except for the *P*_0.5*_ fraction which is overlaid with the parent dispersion. (B) Normalized AFM histograms of all fractions, as well as the corresponding log-normal distribution fits. Despite containing the greatest number of long nanotubes, *P*_0.5*_'s length distribution is more central and broader due to a significant number concentration of short nanotubes observed in the AFM micrographs collected. Note that AUC characterization is not currently feasible for BNNT materials. Sample sizes for AFM length metrology were as follows: *S*_3_: 155, *P*_3_*S*_2_: 295, *P*_2*_S_1_: 389, *P*_1*_S_0.5_: 96, *P*_0.5*_: 75.

For the BNNT PDLS fraction length characterization we rely solely on AFM measurements, as essentially the entirety of BNNT UV-visible absorbance is outside the wavelength range of our AUC instrument. As presented in Fig. 5 and Table 3, the PDLS separation yields distinct populations that are well-segregated based on BNNT length. Average length for the fractions ranged from 137 nm (*S*_3_) to 620 nm (*P*_0.5*_). The 〈*L*_*N*_〉 for *P*_0.5*_ is somewhat less than that of *P*_1*_S_0.5_ due to an unexpected abundance of short nanotubes intermixed with a significant presence of the longest measured BNNTs. This observation appears to be an effect of correlated fractionation of larger diameter BNNTs into this longest fraction. We can make this observation, because in addition to the BNNT length, the AFM cross-sectional step height was measured across each counted BNNT to determine its diameter. Unlike the SWCNT populations, for which little diameter variation was observed, plotting this step height *versus* length for all AFM-imaged objects in each PDLS fraction (Fig. S5†) = uncovers a positive correlation of increasing length and apparent diameter with each successive fraction. Although this increase is noticeable in contrast to all SWCNT populations, the BNNT fractions also display significantly more polydispersity in diameter (as measured by AFM step height) and a large fraction of the material is essentially of constant diameter separated by length across the PDLS fractions. The larger, differentially distributing, objects are likely “impurities” from a combination of bundling and/or wall number dispersity in these multiwalled nanotubes. Overall, from the observed increase in particle diameter with successive PDLS stage and the early-stage reduction in non-rodlike particles, fraction *P*_2*_S_1_ is likely the least polydisperse of the resolved BNNT populations.

Although the UV-vis-NIR absorbance of BNNT materials is very different from that of SWCNTs, several notable corroborating features also appear in the absorbance spectra shown in Fig. 5B. Firstly, fractions *S*_3_ and *P*_3_*S*_2_ have prominent low-energy shoulders at ≈5.9 eV distinct from the main absorbance feature at 6.1 eV. It is not definitive, but this peak could correlate to the presence of defective and non-nanotube material in these fractions; these were seen in AFM micrographs as isotropic particles less than 100 nm in size and became markedly less prevalent in images of latter fractions (Fig. S11†). It is also possible that such particles are fragments of hexagonal boron nitride (h-BN) flakes/sheets. The wavelength of the primary B–N absorbance peak at ≈6.1 eV is also observed to gradually redshift across the separated length fractions, shifting from 203 nm to 205 nm in longer fractions. A hypothesis for this observation could be a change in the average dielectric environment experienced for the carriers confined within individual BNNTs, perhaps from the increasing and possibly nonuniform wall number. However, this is unclear as the intrinsic BNNT dielectric constant should be less than that of the adsorbed DOC layer. A second possibility is that DOC adsorbs in a more porous layer structure with increasing BNNT diameter, leading to redshifts from a greater surface accessibility by water.

In a practical observation, for the fractionation of BNNT populations by PDLS it was noticed that a substantially greater proportion of the long nanotube total mass was pelleted in a single fractionation. In other words, recycling steps only propagate minimal additional BNNT mass. We hypothesize that this is due to the greater density and absolute, per length, mass of BNNTs, both of which increase expected sedimentation rates for individual or clustered BNNTs relative to those of SWCNTs or to any disruptive convective flow in the centrifuge tube. The impact would be to yield a greater probability of successful translation into the pellet phase of a polymer depletion-driven nanotube cluster, remembering that in neither case is the magnitude of the centrifugal acceleration sufficient to deplete a well-individualized particle on the time scale of the applied centrifugation. Alternatively, it could be a number concentration effect, as there are fewer BNNT particles than SWCNTs at the same mass concentration, and absolute effectiveness of fractionation can vary due to phase diagram effects.^55^ Lastly, recent computational and empirical findings of improved mechanical resilience and strength of BNNT–polymer composites as compared to those of CNTs might alternatively suggest an interplay with the PMAA polymer due to stronger enthalpic BNNT surface–polymer interactions,^55,56^ affecting, *e.g.*, the average size or cohesiveness of the depleted clusters in a manner distinct from the SWCNTs. Study of these effects, however, is beyond the scope of this contribution.

### Versatile approaches for high-resolution refinement of length distributions

**Fig. 6:**
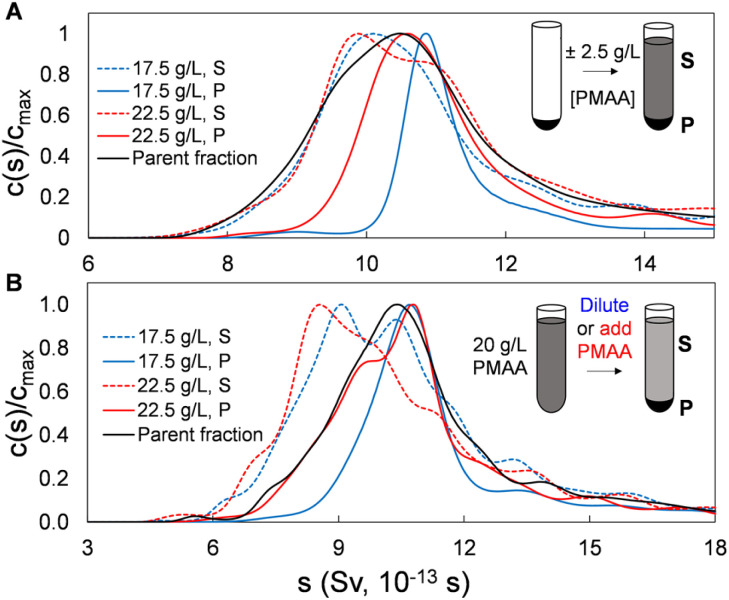
Extending the limits of PDLS length resolution. AUC sedimentation coefficient profiles demonstrating further length-specific enrichment in both precipitates and supernatants of a SG SWCNT nanotube fraction through a single additional separation stage. The parent nanotube material originally PDLS separated with a 20 g L^−1^ PMAA solution was re-separated *via* (A) resuspension of the precipitate at slightly modulated polymer concentrations, or (B) directly modifying the PMAA concentration of the first step supernatant.

To this point we have demonstrated and characterized the successful separation of both SWCNT and BNNT nanotube populations into significantly length-narrowed fractions. We now demonstrate that such fractions can furthermore be directly reprocessed using the same PDLS method to further narrow the achieved length distributions by re-separation at polymer concentrations close to those of their original fractionation condition. This is reasonably equivalent to making a smaller polymer concentration step size in the initial fractionation but is preferable on a process level due to the observed separation efficiency of each PDLS step. As shown below, this re-PDLS methodology enables a match to, or improvement in, the narrowness of the distribution width as compared to chromatography-based length separation methods. Notably, such enhancement to the separated population distribution can be applied to any stage or fraction from the PDLS separation process (either precipitate or supernatant). Moreover, unlike methods involving stationary or porous media that may lead to irretrievable deposition/loss of nanotube mass, we observe that nanotubes are fully recoverable through PDLS, and for any segment of the length distribution, simply by dilution with polymer-free dispersant solution. Results of an example adaptable refinement scheme are shown in Fig. 6 for SG SWCNTs separated by two manners of finely modulated polymer concentrations. In this plot, the sedimentation coefficient distributions shown for concentration modulations of (±2.5 g L^−1^ PMAA of the previous concentration), applied to both (a) sedimented and (b) dispersed nanotube fractions, demonstrate significant distribution narrowing by a single additional PDLS step.

For this experiment, the starting population were single-step (no recycling) PDLS *P*_2_ and *S*_2_ fractions directly from the original parent (*i.e.*, by PDLS with 20 g L^−1^ PMAA). For the *P*_2_ fraction, the refinement process is performed by re-PDLS at either 17.5 g L^−1^ and 22.5 g L^−1^ PMAA. Similarly, the PMAA concentration of the supernatant fraction was modified by either dilution with PMAA-free 10 g L^−1^ DOC solution, or by addition of a small aliquot of high concentration PMAA (Fig. S15†). Significant macroscopic nanotube partitioning was observed in all cases, demonstrating the sensitivity and relative selectivity of the process for even small PMAA concentration increments. That re-fractionation reduces dispersity of all precipitates is shown in the AUC results in Fig. 6, with the 17.5 g L^−1^ PMAA concentrations, as expected, resulting in the narrowest distributions. As a note, care must be taken to prepare any parent material as being rid of co-precipitated bundles and aggregates by preventatively applying a low concentration of polymer (*e.g.*, 2 g L^−1^), and/or subjecting the dispersion to a brief (seconds to a minute) period of tip ultrasonication to individualize associated nanotubes if re-fractionation is not performed directly following a previous PDLS separation. Conversely, the supernatant separated with 22.5 g L^−1^ PMAA for either parent dispersions yielded appreciable retention and population enhancement of the shortest nanotubes. Notably, most of the nanotube mass is retained in the supernatant for both precipitate resuspension and “direct” methods.

If the number of independent PDLS steps becomes too cumbersome for fine fractionation, one may consider simply integrating SEC fractionation into the workflow (*e.g.*, after several PDLS steps) to more finely resolve a given length populations in a single process step. This can be readily achieved as long as limited mass throughput is not a concern, and the depleting polymer is removed from the dispersion prior to SEC.

### Effect of processing conditions on separation fidelity and yield

In addition to including a recycling step to increase mass throughout and improve supernatant fraction fidelity, we also evaluated the effect of settling time and SWCNT concentration on the PDLS process in a single fractionation stage. For these experiments, parent SG SWCNT material was dispersed in 20 g L^−1^ PMAA and allowed to settle from (1 to 7) days at three SWCNT concentrations: (0.05, 0.125, and 0.25) mg mL^−1^; this PMAA concentration was expected to yield a roughly 40 : 60 split of the mass between supernatant and precipitate. Significant changes in yield (*i.e.*, partitioned mass into the pellet with a single centrifugation) for the two greater SWCNT concentrations were observed up to 5 d after dispersion, at which point the absolute mass was found to be evenly split (≈50 : 50) between the supernatant and pellet for these specific samples. Such differences are observed, however, to be a function of the absolute nanotube concentration. For the 0.05 g mL^−1^ SWCNT concentration case, the precipitated mass increased monotonically to a total, absorbance measured, value of 63% after 7 d of settling, exceeding the yield into the pellet phase found in the more enriched dispersions (Fig. S16†). This highlights complexity in partitioning phase boundaries that are poorly mapped; the dispersed rod volume fraction however has previously been identified in determining the phase boundary in depletion, and more dilute rod concentrations were reported to encourage homogeneous microscopic clustering.^57^ Such a phase boundary shift for decreased particle concentration would act to increase mass yield at lesser nanotube concentration of a recycling step, and may be a partial source of the success of the two-step PDLS scheme used in this contribution. Worth noting is that no significant changes in the length distribution of the supernatant fraction was observed by AUC characterization from samples spanning the first 5 d of incubation. This indicates that the length-dependent partitioning is not kinetically limited, and that, the likely explanation for a continued increase in precipitated mass is such a shifting partitioning phase boundary with nanotube concentration.

### Effect of nanotube diameter on depletion effects

**Fig. 7:**
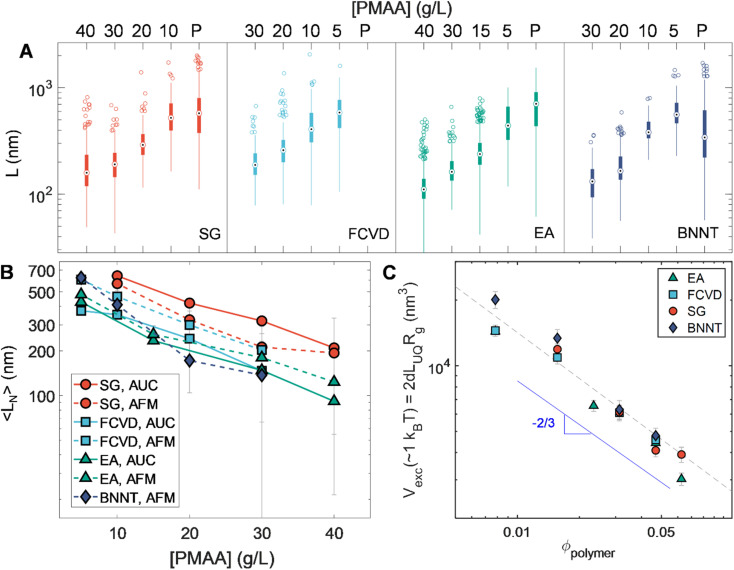
Summary and analysis of PDLS results. (A) Box plot summary of the AFM-measured lengths of nanotubes within each separated fraction. The thick bar envelops the central 50% of measured nanotube lengths (inter-quartile range, or IQR), the whiskers extend to 1.5 × IQR away from each end of the bar, and any depicted points outside of this range are defined as outliers. Note the complete predominance of long nanotube outliers owing to the “reverse sequential” separation conducted. (B) Number-averaged lengths (AFM- and AUC-derived) of each fraction plotted against applied polymer concentration of depletion. The clear trend of increasing nanotube length from highest to lowest SWCNT diameter for the same polymer concentration suggests a dependence of excluded volume on nanotube diameter. (C) Excluded volume *versus* polymer volume fraction for nanotubes calculated by noting the nanotube length thresholds at which partitioning occurs for a given polymer concentration (near upper quartile for each separation stage, *L*_UQ_) and scaling by the hydrodynamic diameter (*V*_exc_ ∝ *L*_UQ_*d*_h_).

An additional set of observations is enabled by the breadth in diameter of the nanotube populations used in this contribution. These are developed in Fig. 7. Fig. 7A summarizes the length statistics obtained from AFM for all sorted nanotubes. Plotting number-averaged length, 〈*L*_*N*_〉, against applied polymer concentration (Fig. 7B) illustrates a trend of increasing length for a given depletant concentration with decreasing SWCNT diameter. Given the entropic basis of our methods, this likely arises from diameter-dependent differences in excluded volume for the same length of nanotube. As excluded volume is expected to scale with the volume of the depleted colloid itself, Fig. 7C depicts the calculated occupied spherocylindrical nanotube volume for each of the different nanotube populations against the polymer volume fraction (*ϕ*_polymer_). *ϕ*_polymer_ was calculated by dividing the mass per volume polymer concentration by the molar mass (6 kDa) and calculating a unit volume by assuming a coil in theta solvent with a radius of gyration (*R*_g_) of ≈1.504 nm.^43^ Having shown that the length partitioning point is not kinetically determined, we assume the association energy to deplete a particular length of nanotube is ≈1*k*_B_*T*. However, the average length of a population is not the correct value for these volume calculations as it is affected by the distribution of lengths existing in its immediate parent population. Instead, excluded volumes were calculated from apparent threshold lengths for partitioning at each applied polymer concentration, which we take for convenience as equivalent to the upper quartile length value (*L*_UQ_) of each distribution, on the basis that this value will more closely reflect the cutoff length of selective partitioning. These values were then multiplied by the nanotube diameter-dependent hydrodynamic diameter (*d*_h_) values from Table 1 to determine the excluded volume. For spheres, the depleted sphere diameter scales inversely with polymer concentration; empirically, we observe a scaling dependence of roughly *ϕ*_polymer_^−2/3^. Given the additional orientational dependence of depletion effects in rods, there is likely to be a relationship between local degrees of nanotube alignment and excluded volume affecting the scaling.^58,59^ Compellingly, the distribution of orientational states is mediated by rotational diffusivity which scales as L^−3^. However, future work and experiments designed to yield depletion length thresholds at greater precision will be necessary to discriminate between any particular hypothesis for the observed scaling. If it is rotation derived, experiments on smaller diameter (and thus flexible) SWCNTs in which bending decreases the effects of length may be particularly valuable in the discrimination.^46^

The empirical universal scaling however offers a practical estimation of the partitioning length threshold for other nanotube sources or separated (*n*, *m*) samples as long as the nanotube hydrodynamic diameter is known or can be estimated. To this purpose an empirical equation is provided in the ESI† for straightforward guidance in targeting of arbitrary length separations. This enables practical implementation of PDLS while the physical source of the observed scaling is determined in future study.

### Utility of SWCNTs as a model system

The fractionated SWCNT populations of this work are also potentially useful as model materials for studying dispersed anisotropic particle behavior. Most current model rodlike or filamentary colloids are of biological origin (*e.g.*, tobacco mosaic virus,^60^*fd*-virus,^61^ actin,^62^ double stranded DNA^63^), which provides limited experimental choice in independently selecting rod length, diameter, and persistence length for experimental studies. While some other promising synthetic analogues exist,^64^ their tunability with respect to size and mechanical properties remains similarly limited. The highly length-refined nanotube populations in contrast are significantly tunable through material selection and may extend the feasible suite of characterization methods (*e.g.*, light, neutron, and X-ray scattering) for observing *in situ* solution-state association behavior.

Aside from providing more refined rodlike systems for study, our findings spur further open questions about the dynamics of the association process. These include determination of the true excluded volume by nanotubes as a function of length, and confounding effects limiting approach to equilibrium, *i.e.*, the extent to which polymers occupy the scribed volume of nanotubes in the isotropic/dispersed phase. Both likely involve analysis of competing rotational and translational diffusion timescales for the particle and depletant, as well as a timescale relative to collective many-body association. Such interaction will be complicated by flexibility. Another direction of study could be whether enthalpic interactions can be used to further control length partitioning, *e.g.*, improved formation of aligned domains, or to induce selective depletion effects.^65,66^ Another interesting question is whether, in the course of partitioning, do shorter nanotubes also themselves begin to act as rodlike depletants that further drive the approach and coarsening of nanotube assemblies?^59,67,68^

### Practical processing considerations

The simplicity and minimal equipment required for PDLS suggest significant scalability and versatility for its application across nanotube research and in support of applications. Viable kilodalton-sized depletants such as PMAA, polyethylene glycol (PEG), and sodium–poly(styrene sulfonate) (PSS) are readily available and may be varied in molecular weight to probe or further control depletion forces.^38^ Aside from the nanotube material itself, in our use the anionic surfactant is the dominant chemical cost across the entire PDLS process. Repeated multi-stage PDLS process should thus be economically feasible, with added control over or choice of the target mass distribution or length resolution adding product value at each fractionation step. This is in contrast with chromatography and flow-based methods, where columns and separation phases introduce significant (consumable) equipment costs, flow channel dimensions intrinsically limit throughput, length resolution is generally prescribed to nanotubes <1 μm, and dispersion concentrations are limited for effective separation. A comparative chart is presented in Table 4. For PDLS, given enough quiescent settling time even a large separation funnel can fractionate appreciable amounts of precipitated material. Thus, although centrifugation speeds the process, centrifuge vessel volume is not a bottleneck for mass throughput. Obtained pellet-phase length fractions are also highly concentrated, facilitating easily transfer to an aqueous DOC solution environment for final use or propagation into other aqueous processing (*i.e.*, ATPE-based metallic/species sorting).

**Table 4:** Comparison of nanotube length-sorting techniques, along with the implicit tradeoffs in complexity, time, resolution, and material/equipment needs

Method	Length resolution	Time for one process (h)	Media	Equipment involved	Approx. # of post-process fractions	Advantages	Disadvantages
Ultrasonication^23,69^	Poor, except at long times and short nanotubes	≈(4–48)	Parent suspension	Sonicator	1	No additional equipment needed	Shortening only, defect induction
Differential sedimentation^70^	≈Factor of 2 in length	≈1	Parent suspension	(Ultra)centrifuge	2	Easy	Poor resolution, single fractionation per step
Ultra-centrifugation^34,71,72^	≈30% of 〈*L*〉 per fraction	≈(4–72)	Density gradient medium	Ultracentrifuge	>10	Concomitant defect reduction in some fractions	Low mass throughput, requires DGU media, slow
PDLS (ref. 38, 39 and this work)	≈±50 nm	4–24	Aqueous polymer solution	Centrifuge (optional, increases resolution)	2	Easily controlled fractionation points, no mass throughput limit	Relatively slow process, single fractionation per step, requires polymer addition
Gel chromatography^32^	±150 nm (BNNTs)	≈(1 – 3)	Porous gel stationary phase	LC system, gel column	>10	Rapid and multiple fractionations in a single step	Mass throughput limitations, risk of mass loss
HPLC-SEC^31,40,73^	≈±30 nm	≈1	Packed columns of porous stationary phase	HPLC system, porous media column(s)	>10	Rapid and multiple fractionations in a single step	Mass throughput limitations, risk of mass loss, costly columns

### PDLS as a simple purification method

A separate practical implementation is suggested by the observed differential fractionation of rod-like and non-rod particles in the results above. Small and non-rodlike impurities can be common in typical centrifugation-only purified SWCNTs because their sedimentation rates will be less than those of the SWCNTs for non-dense (*i.e.*, non-catalyst) particles and thus not removed by the process. Adding a PDLS step to remove such particles can complement or replace (with lesser achieved purification) separations such as rate-zonal ultracentrifugation, even if minimal length fractionation of the parent dispersion is desired. For example, a single step of 40.0 g L^−1^ PMAA PDLS to multiple types of SWCNT soot sources is observed to significantly remove non-peak UV-vis-NIR absorbance from the spectra of SWCNTS fractionated into the pellet fraction, indicating an effective removal of non-SWCNT material. An example of this effect as observed for a non-rate zonal ultracentrifugation separated SWCNT dispersion synthesized by a high-pressure carbon monoxide process (HiPCO), before and after PDLS, is reported in the ESI (Fig. S18†). The reduction in “background” absorbance is approximately 50% of that obtained through rate-zonal purification, but with much reduced equipment specifications and chemical costs (*e.g.*, avoiding the cost of density gradient media). These findings suggest that raw nanotube dispersions stand only to benefit from a preliminary application of depleting polymer, whether for impurity removal or size refinement, and that PDLS should be considered as a processing staple in the cascade of aqueous-phase nanotube purification and separation.

## Conclusions

We have extended the range of applicability for depletion-based length separation of various SWCNT types as well as BNNTs, at various degrees of material purity and refinement. Through rigorous cross-comparisons between single- and large-ensemble length metrology, nanotube partitioning was found to depend on the diameter of the particles being depleted, though a general excluded volume scaling was found to predict separation of target lengths with applied polymer concentrations. Application of multiple separation stages can achieve length resolutions rivalling those of chromatography methods, separation can be feasibly scaled to arbitrary quantities and high yield with precipitate recycling, and the resulting fractions may be integrated and combined with other aqueous processing workflows such as ATPE, ultracentrifugation, or SEC. We anticipate this contribution to be of interest and importance to experimentalists working broadly with 1-D nanomaterials, as well as theoreticians looking to cross-validate computational findings in both nanotube-specific and broader rodlike colloidal systems.

## Materials and methods

Certain equipment, instruments, software, or materials, commercial or non-commercial, are identified in this paper in order to specify the experimental procedure adequately. Such identification is not intended to imply recommendation or endorsement of any product or service by the National Institute of Standards and Technology (NIST), nor is it intended to imply that the materials or equipment identified are necessarily the best available for the purpose.

### Materials

Sodium deoxycholate (DOC, BioXtra >98%, Sigma), iodixanol (sold as Opti-Prep, 60% mass/vol solution, Sigma), heptane (C_7_H_16_, EMD Millipore), tetracosane (C_24_H_50_, Aldrich), deuterium oxide (D_2_O, 99.8%, Cambridge Isotopes) and poly-methacrylic acid (PMAA, (4 to 6) kDa, Aldrich) were purchased from commercial vendors and used as received. Ultrapure water was collected from a Barnstead MicroPure system. Nanotube materials were obtained from commercial vendors as follows: electric-arc discharge (EA) synthesized SWCNT soot (Carbon Solutions, P2 grade lot A011), (6, 5) enriched cobalt-molybdenum catalyst (CoMoCAT) synthesized soot (Chasm Nanotechnologies SG65i-L64), floating catalyst vapor deposition method (OCSiAl, Tuball lot 01RW02.N1.370), and refined BNNT puffballs (BNNT, LLC, batch BNNT B, Lot Y5B01220211B).

### Parent nanotube dispersion and processing

Prior to aqueous dispersion, EA SWCNT soot was soaked in liquid heptane for ≈24 h, then filtered against filter paper and allowed to fully dry.^42^ This process fills the interior, endohedral, volume of the nanotube with the heptane, which in concordance with literature we describe with the terminology C_7_H_16_@EA SWCNT. The filter cakes were dispersed with tip ultrasonication (Fisherbrand FB120, tip = 6 mm at 93% amplitude yielding ≈0.9 W mL^−1^ applied power) for 45 min in an aqueous solution of 20 g L^−1^ sodium deoxycholate (DOC), at a nominal nanotube mass loading of ≈ 1 mg mL^−1^. Gross impurities were removed by centrifugation in a JA-20 fixed angle rotor in a J2-21 high speed centrifuge (Beckman) for 2 h at 1884 rad s^−1^ (18 krpm), collecting the supernatant. Structurally pristine, fully individualized, alkane-filled nanotubes were isolated from this parent dispersion *via* rate-zonal ultracentrifugation (RZU) in a Beckman L80-XP ultracentrifuge with a VTi65.2 or VTi50 rotor using a 10% iodixanol (mass/volume%) as the race layer as in previous reports.^41^ The purified top RZU band was chosen for further sorting and rid of iodixanol by exchanging into an aqueous 10.0 g L^−1^ DOC solution by stirred-cell ultrafiltration using either a 30 kDa or 100 kDa molecular mass cutoff regenerated cellulose membrane (Millipore).

Small diameter nanotubes (SG SWCNTs) synthesized by the CoMoCAT method and moderate FCVD Tuball (≈1.3 nm diameter) populations were dispersed in an aqueous 20 g L^−1^ DOC solution by ultrasonication followed by centrifugation and collection of the supernatant. RZU was similarly performed as for EA SWCNTs, but with 9% or 10% iodixanol with 10 g L^−1^ DOC race layers, respectively, collecting the main band containing the structurally purified SWCNTs. Similar to the EA SWCNTs, FCVD SWCNTs were filled with a linear alkane (in this case C_24_H_50_) prior to the dispersion process. For this filling, the alkane and FCVD nanotube soot were incubated together at ≈60 °C, which is above the melting point of the alkane, for 24 h prior to filtration against a 0.1 μm pore size VVLP membrane and rinsing with heptane to remove externally absorbed alkane molecules.^42^ For the C_24_H_50_@FCVD SWCNTs, aqueous two-polymer phase extraction (ATPE) was performed on the RZU separated dispersion as previously reported to further purify the SWCNT population.^74^ First, a highly metallic nanotube species enriched daughter fraction of the C_24_H_50_@FCVD SWCNTs was isolated,^75^ followed by a second diameter-based separation fractionation cascade to reduce the diameter polydispersity of the final sample.^26,74^ As-received BNNT material was dispersed in aqueous 20.0 g L^−1^ DOC with 35 min of ultrasonication. The resulting dispersion was then centrifuged at 838 rad s^−1^ (7.4 krpm or 8000*g*) and the supernatant reserved as the parent dispersion for length sorting.

### Length separation

Length separation was conducted with the reverse sequential precipitation method, used as described in Gui *et al.*,^39^ with a longer settling time after each polymer application and a precipitate recycling step to maximize nanotube mass carryover and separation yield in subsequent stages. The procedure is schematically depicted in Fig. 1B. First, nanotube parent dispersions (10.0 g L^−1^ DOC in deionized H_2_O) were introduced into the PMAA solution, arriving at a tube concentration in the range of (0.1 to 0.25) mg mL^−1^. The resulting mixtures were allowed to sit at ambient laboratory temperature (≈22 °C) for 24 h and centrifuged thereafter at 1047 rad s^−1^ (10 krpm, and 1309 rad s^−1^ or 12.5 krpm for SG SWCNTs) for 10 min. The precipitate (*P*_*X*_) was re-dispersed in a small amount of 10 g L^−1^ DOC solution, while the supernatant was transferred to a secondary tube that was allowed to sit quiescently for an additional 24 h. This supernatant was then re-centrifuged; this secondary precipitate 
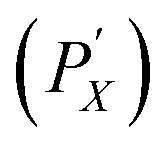
 was resuspended and added to *P*_*X*_, which proceeded to the next separation step while the supernatant (*S*_*X*_) was reserved. This process was repeated for a total of four stages with decreasing PMAA concentrations (concentrations were adjusted for each tube type to achieve well-spaced tube length partitions; see Table S2 in the ESI†). It was empirically determined that the supernatants settle completely up to one week after initial polymer application, likely owing to mass-action effects as longer nanotubes are continuously removed from the bulk dispersion.

### Absorbance spectroscopy

Prior to absorbance measurements, all samples were reset to a surfactant concentration of 10.0 g L^−1^ DOC and washed of polymer through successive concentration and dilution in a stirred ultrafiltration cell. These were measured through a 1 mm path quartz cell using a Varian Cary 5000 spectrophotometer with a 1 nm step and a 2 nm bandpass. Subtraction of separately measured 10.0 g L^−1^ DOC reference spectra was performed to remove the contributions of water and DOC from the spectra during data analysis.

### Atomic force microscopy

Atomic force microscopy was carried out on a Bruker Dimension Icon system in peak-force tapping mode. Sample preparation for atomic force microscopy was carried out as detailed in Silvera Batista *et al.*^40^ Silicon was functionalized with APDMES as an imaging substrate. Stock nanotube dispersions were diluted at least 500× in 2 g L^−1^ SC and 20 mM NaSCN; single-microliter amounts were incubated on the wafer for (5 to 30) min, before rinsing with DI H_2_O, depending on the initial stock nanotube concentration. Microscopy was conducted on a Bruker Dimension Icon atomic force microscope, in tapping mode, at increasing scan sizes with longer nanotube fractions (AFM scan edge lengths: [4, 6, 8, or 10] μm). Nanotube length and step height (as a semi-quantitative proxy for nanotube diameter) was determined with a semi-automated MATLAB script that background-subtracted, threshold flattened, and skeletonized user-defined image segments containing an individual or nearly-individualized nanotube. Step height was obtained *via* a root-mean-square of the height within the thresholded bright pixels, while length was redundantly confirmed *via* both Euclidean distances between nanotube ends and contour lengths obtained from weighted curve fitting.

### Electron microscopy

Solid samples of each parent nanotube source were prepared by hydrating a pinch of powder with isopropanol and drop-casting onto EM grids with an ultrathin carbon film on a lacey carbon support. For post-PDLS liquid-phase samples, an aliquot of dispersion was drop-cast directly onto a grid and allowed to dry. Each grid was then rinsed multiple times by dropping on isopropanol and wicking the fluid off the grid.

A Zeiss Gemini 300 Variable Pressure scanning electron microscope (SEM) was used for electron imaging. Sample images were recorded with the in-lens detector using a primary electron beam energy of 10 keV.

### Analytical ultracentrifugation

AUC was conducted in a Beckman–Coulter XL-I analytical ultracentrifuge with an AnTi-50 rotor and standard 12 mm optical pathlength cells with sapphire windows at a rotation rate of 2932 rad s^−1^ (28 krpm). Both absorbance and interference detector trains were recorded, but absorbance-based detection at the primary UV peak (anhydrous measurements) or visible wavelength peak transition (buoyant density measurements) was utilized for analysis due to a greater observability of the sample signal. An initial nanotube absorbance of ≈0.25 A through the 12 mm cell was targeted for fractions containing short nanotubes and even more dilute for longer samples to avoid notable deviation from ideal sedimentation behavior readily observed for extended colloids.^76^ The temperature was 20.0 °C and ensured by a minimum of 1.5 h of temperature equilibration. The density and viscosity of the exact 10.0 g L^−1^ DOC solutions at 20.0 °C were measured using an Anton-Parr DMA 5000 – LOVIS M densitometer–viscometer. Analysis of the recorded radial absorbance profiles as a function of time was conducted using the numerical fitting software SEDFIT V16.1c.^77,78^ Sedimentation was modelled using the *c*(*s*) model; the meniscus, noise and friction coefficient were fit for each experiment. Conversion of measured sedimentation coefficient distributions to length distributions was conducted using the hydrodynamic models for cylinders and the measured solution and SWCNT parameters.^16,40,79^

### Birefringence microscopy

Settled, intermediate nanotube supernatant fractions of FCVD and SG SWCNTs (*P*_3_*S*_2_ and *P*_4_*S*_3_, respectively) were re-mixed (concentration ≈ 100 μg mL^−1^), and 10 μL of each was deposited on a glass slide with a 0.1 mm imaging spacer. The samples were allowed to quiescently form polymer–nanotube aggregates for several days and were imaged with an Olympus BX51 microscope in transmission mode, rotating the stage in a static crossed-polarizer configuration to probe tactoid orientation relative to the illumination polarization axis.

## Data availability

The data supporting this article have been included as part of the ESI† or are directly provided in the text.

## Author contributions

Conceptualization: PS, JAF; methodology: PS, JAF; investigation: PS, BKB, EM, MMN, JAF; visualization: PS; supervision: JAF; writing – original draft: PS, JAF; writing – review & editing: PS, BKB, JAF.

## Conflicts of interest

There are no conflicts to declare.

## Supplementary Material

RA-014-D4RA01883D-s001

RA-014-D4RA01883D-s002
