# Supplementary material for: Universalized and robust length separation of carbon and boron nitride nanotubes with improved polymer depletion-based fractionation
Source: RSC Adv. 2024 Aug 28;14(35):25490–506. doi: 10.1039/d4ra01883d (PMC11353058; doi:10.1039/d4ra01883d)
Supplement: RA-014-D4RA01883D-s001 [file RA-014-D4RA01883D-s001.pdf]

This journal is © The Royal Society of Chemistry 2024

Electronic Supplementary Information for

Universalized and robust length separation of carbon and boron nitride nanotubes with improved polymer depletion-based fractionation

Shapturenka *et al.*

*Corresponding author. Email: Jeffrey.fagan@nist.gov

Official contribution of the National Institute of Standards and Technology
Not subject to copyright in the United States

Certain equipment, instruments, software, or materials, commercial or non-commercial, are identified in this paper in order to specify the experimental procedure adequately. Such identification is not intended to imply recommendation or endorsement of any product or service by the National Institute of Standards and Technology (NIST), nor is it intended to imply that the materials or equipment identified are necessarily the best available for the purpose.

This PDF file includes:

Supplementary Text
Figs. S1 to S18
Tables S1 to S2

Table of Contents:

1. Acronyms
2. Preparation and characterization of parent nanotube dispersions
3. Characterization of separated population length distributions
4. Precipitation process variations and related data

1. Acronyms:

Analytical ultracentrifugation (AUC)
Arbitrary unit (a.u.)
Atomic force microscopy (AFM)
Birefringence (BR)
Boron Nitride nanotube (BNNT)
Cobalt-molybdenum catalyst (CoMoCAT)
CoMoCat method synthesized nanotube population (SG)
Electric arc method synthesized nanotube population (EA)
Floating catalyst vapor deposition synthesized nanotube population (FCVD)
Near infrared (NIR)
Poly-methacrylic acid (PMAA)
polymer depletion length separation (PDLS)
Rate-zonal (RZ) ultracentrifugation sorting
Scanning Electron Microscopy (SEM)
Single-wall carbon nanotube (SWCNT)
Sodium cholate (SC)
Sodium deoxycholate (DOC)
Sodium dodecyl sulfate (SDS)
Ultraviolet (UV)
UV-visible-near infrared (UV-vis-NIR)

PDLS fraction labelling:

P_X = precipitate fraction from a separation stage with a polymer concentration of X %
S_X = supernatant fraction from a separation stage with a polymer concentration of X %
PY = pellet from stage Y

Supplementary Text

In addition to the length distributions determined *via* hydrodynamic analysis of sedimentation coefficients measured from AUC, histograms of observed nanotube length distributions were measured with AFM. Representative AFM images collected for each fraction of each parent nanotube material are presented in Figures S6-S9. Histograms of the counted populations are presented in Figure S10.

AFM measurements enable both the measurement of length and apparent diameter for each nanotube counted in the length distributions presented in Figure S6. The apparent diameter is determined from a root-mean square average of step heights in the center of each nanotube, relative to the apparent local surface height (background subtracted *via* 2-D spline fitting). Two-dimensional plots of step height (as a proxy for nanotube diameter) *versus* length for each nanotube are presented in Figure S4. Due to variations in the surface height these data are best used to identify apparent aggregates for the three SWCNT samples. In BNNT materials some correlated diameter selection appears to occur along with length selection in PDLS.

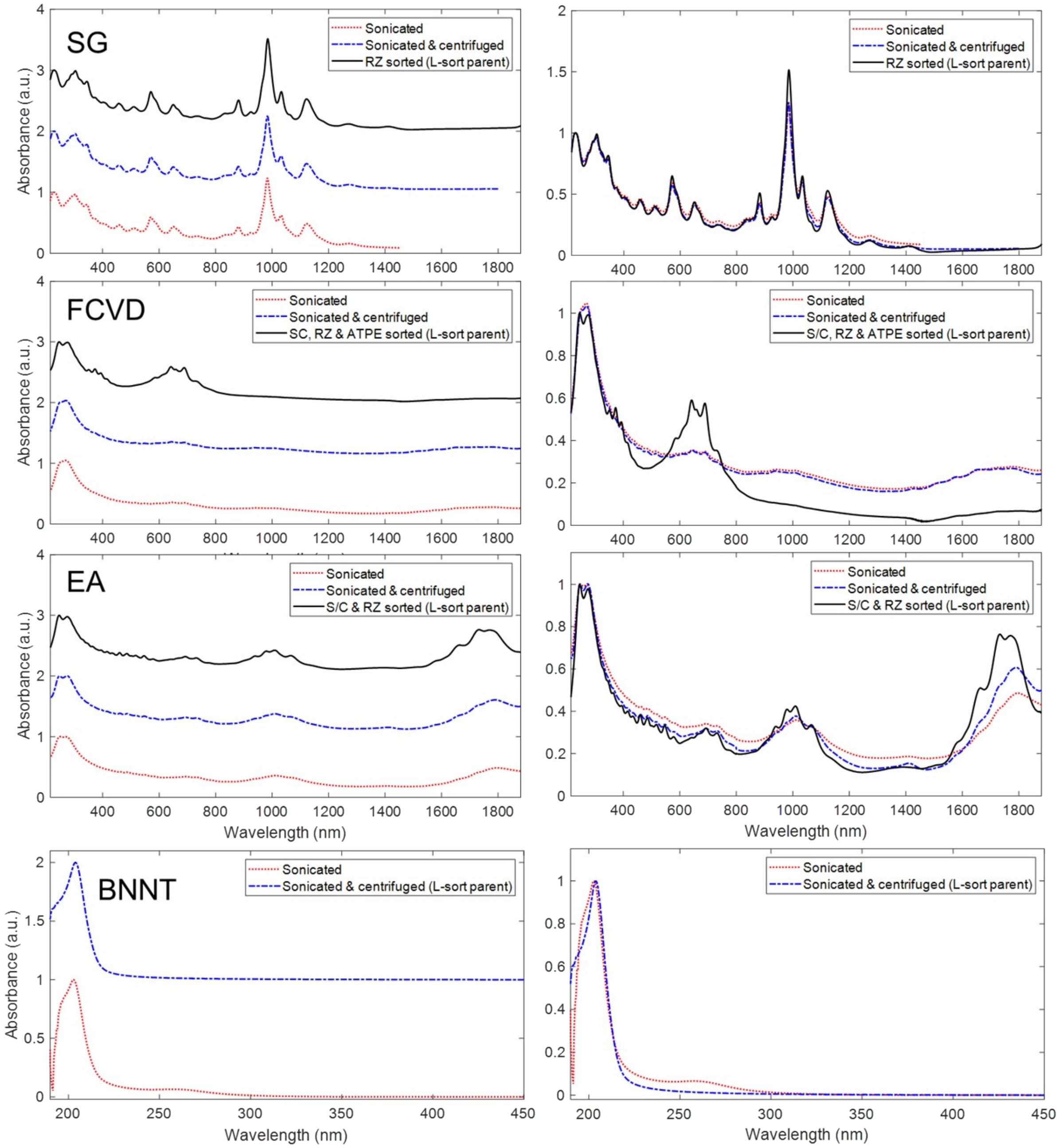

Fig. S1. Preparation and characterization of parent nanotube dispersions.

Scaled UV-vis-NIR absorbance spectra curves for all investigated nanotube materials. Left hand column figures contain scaled spectra at named processing stage offset by 1 arbitrary unit (a.u.); right hand side figures present the same data without an offset. Parent dispersions were processed as follows: SG (CoMoCAT) and EA SWCNTs: sonicated, centrifuged, and rate-zonal (RZ) sorted; (b) FCVD SWCNTs: sonicated, centrifuged, rate-zonally sorted, and ATPE-sorted for both metallic character and a diameter range; BNNTs: sonicated and centrifuged. Each processing step improves the compositional purity of the SWCNTs and concentrates absorbance signal in the remaining peak features.

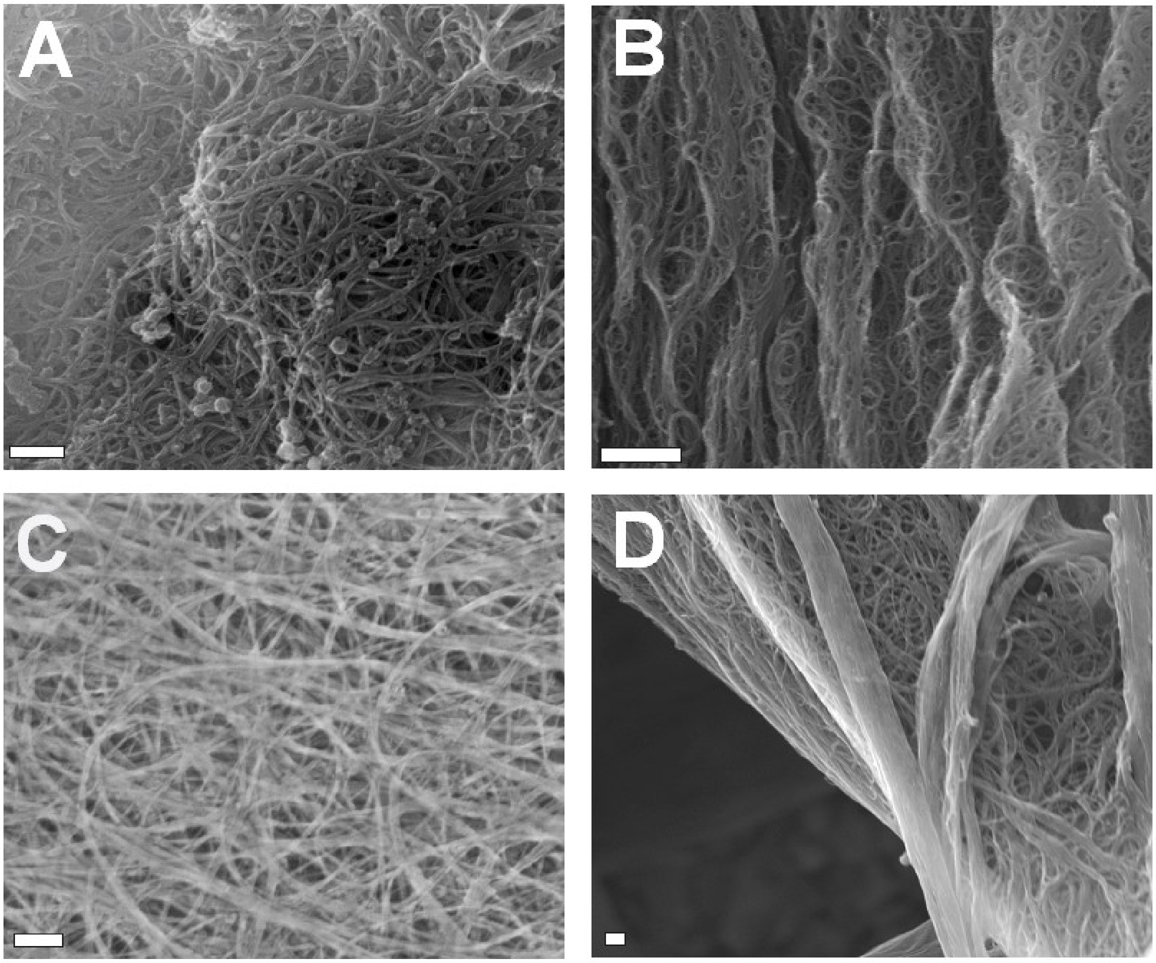

Fig. S2. Electron micrographs of powder nanotube sample materials.

SEM images of the parent nanotube material types investigated in this work. Note that these images are of the materials before dispersion and subsequent dispersed phase purification. (A) Electric arc discharge (EA) SWCNTs. (B) CoMoCAT SG SWCNTs. (C) BNNT. (D) FCVD SWCNTs. Scale bars are 200 nm. While qualitatively demonstrating morphological purity of the samples (low prevalence of non-nanotube material), these images are unsuitable for length determination due to the highly aggregated nature of the material under any viable sample preparation strategy.

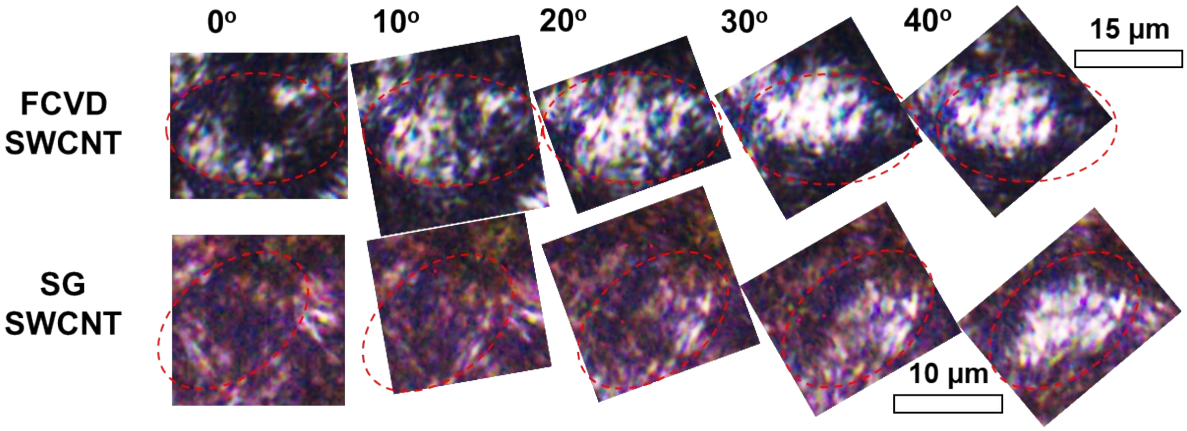

Fig. S3. Microscopic visualization of the depletion process.

Birefringence (cross-polarized) microscopy imaging during a single stage of the PDLS process within sealed fluid volumes demonstrates the spontaneous formation of grouped nematic nanotube tactoids due to the applied depletion. Images are shown for the second fractionation of FCVD and SG SWCNTs at the specified polymer concentration in Table S1. Denoted angles refer to the rotation of the sample cell between the two crossed polarizers of the microscope; 0° was set arbitrarily to the angle at which the tactoid cluster in the center of the image displayed the least light transmission.

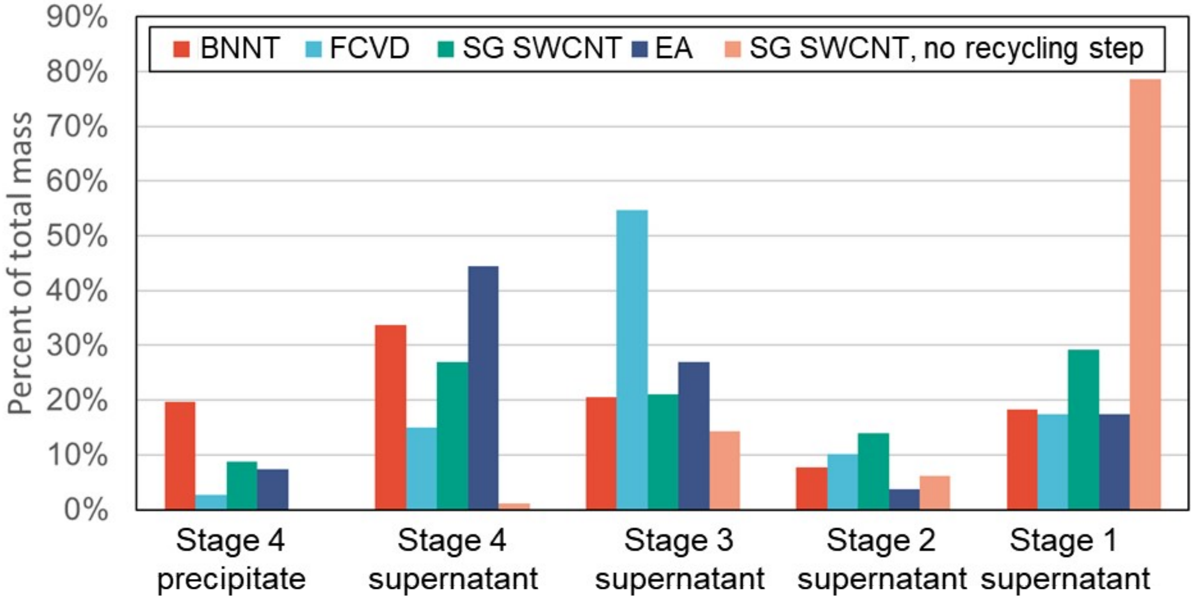

Fig. S4. Tracking mass yields by separation stage and nanotube type.

Distributions of nanotube mass across various separation stages for all nanotube types considered. Two separate SG SWCNT separations are shown to compare forward mass propagation to later stages, one incorporating a 2nd centrifugation after additional incubation (recycling) step (green, middle bar) and one without (peach, far right).

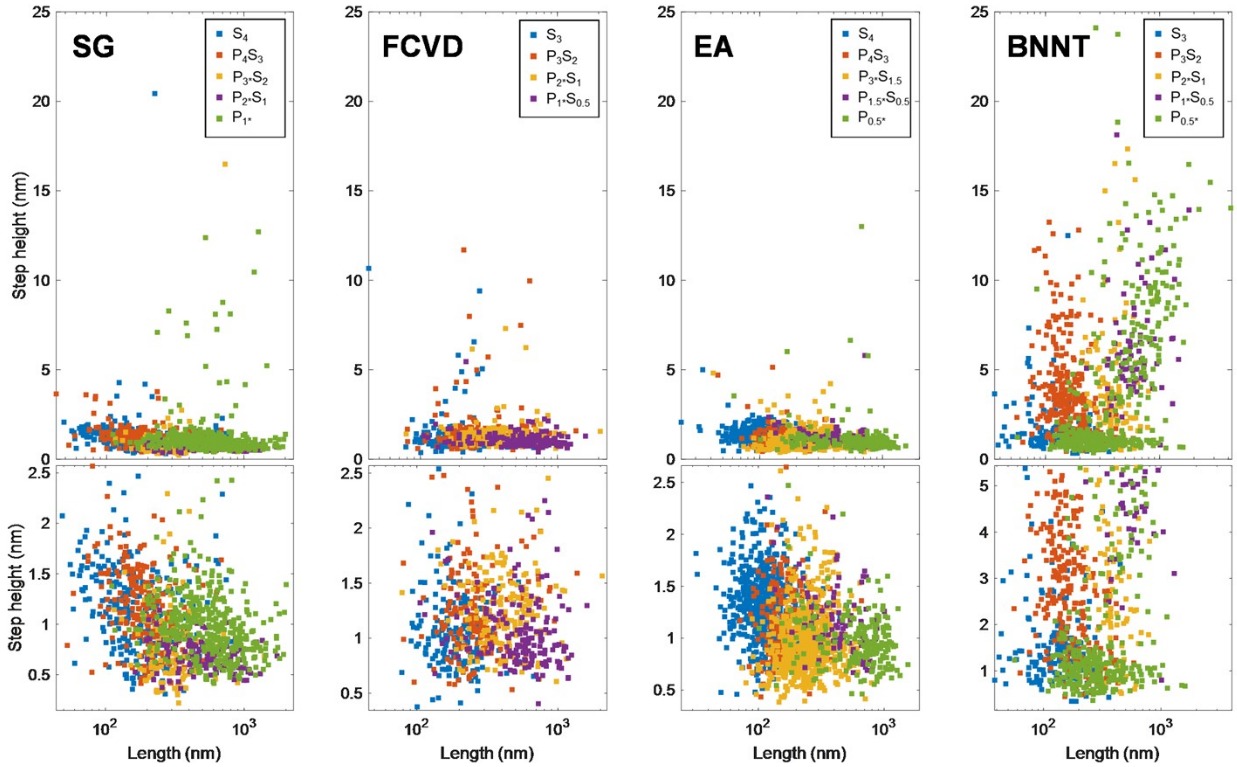

Fig. S5. Characterization of separated population length distributions.

Scatter plots of step height *versus* length of imaged features in AFM for each nanotube type.

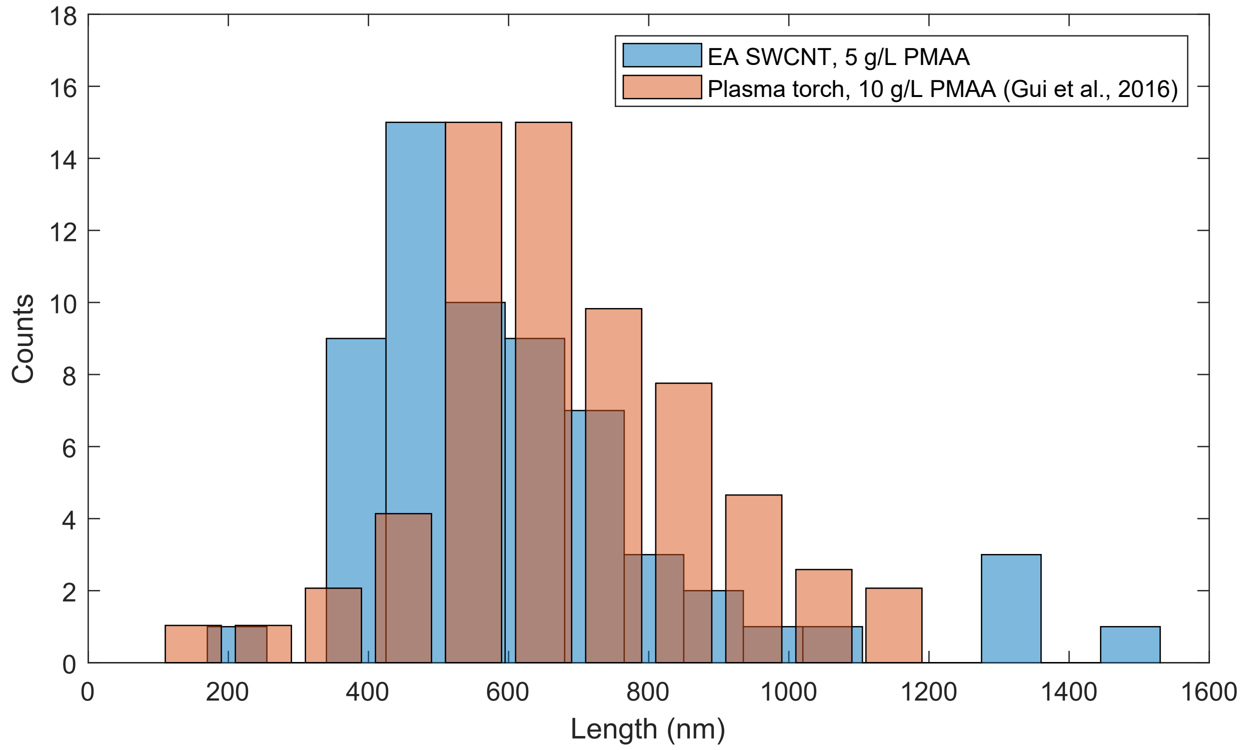

Fig. S6. Length distribution comparison to prior reported PDLS procedure.

Comparison of AFM length histograms from an EA SWCNT length fraction ("$P_{1.5}S_{0.5*}$", 5 g/L PMAA) compared and a plasma-torch SWCNT length fraction precipitated with 10 g/L PMAA [data from Gui *et al.*, Nanoscale (2016)].

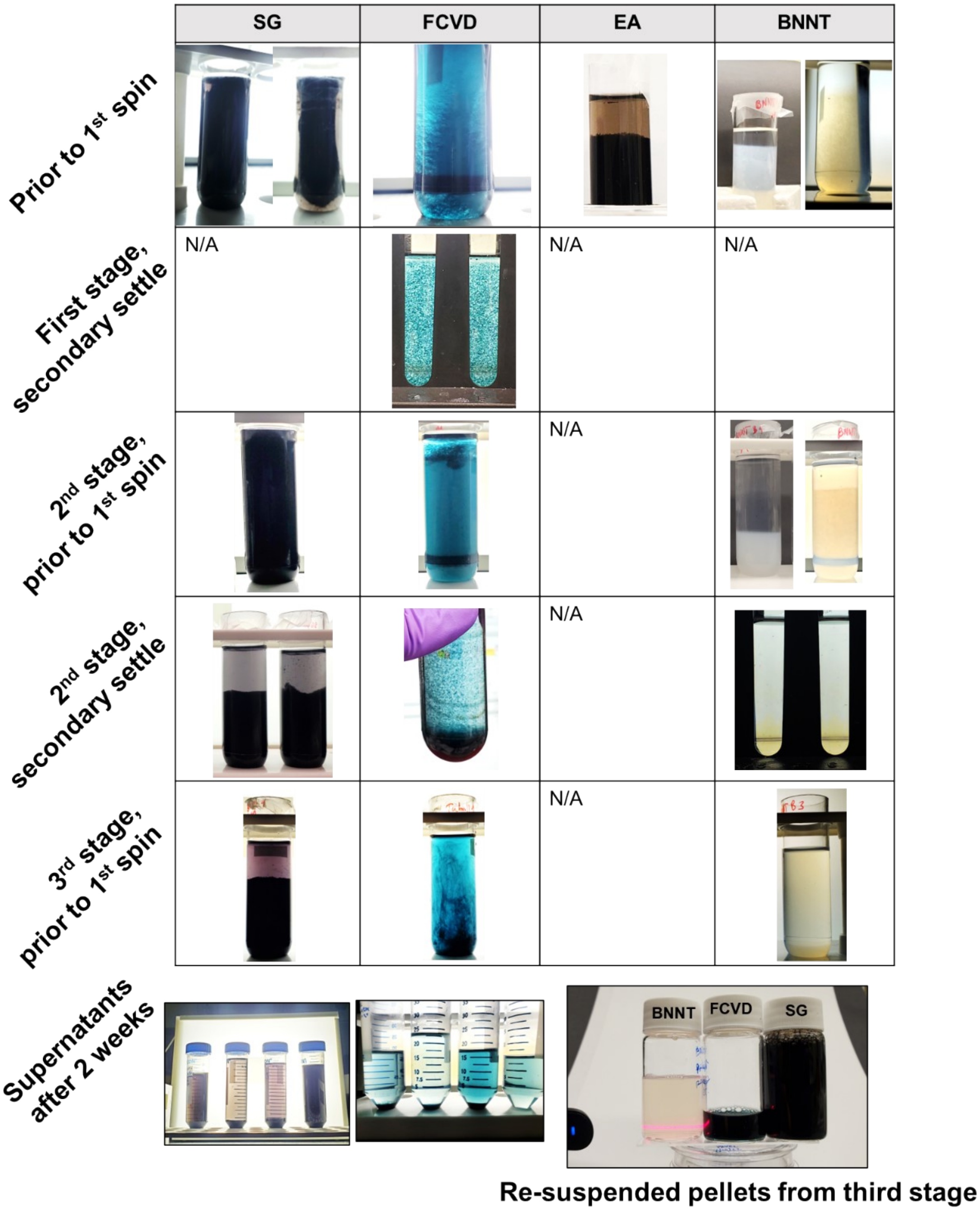

Fig. S7. Photographs of PDLS aliquots during processing.

Photographs of the precipitated mass settling between separation stages for the various nanotube types considered, as well as images of all supernatants after two weeks and collected P3 precipitates.

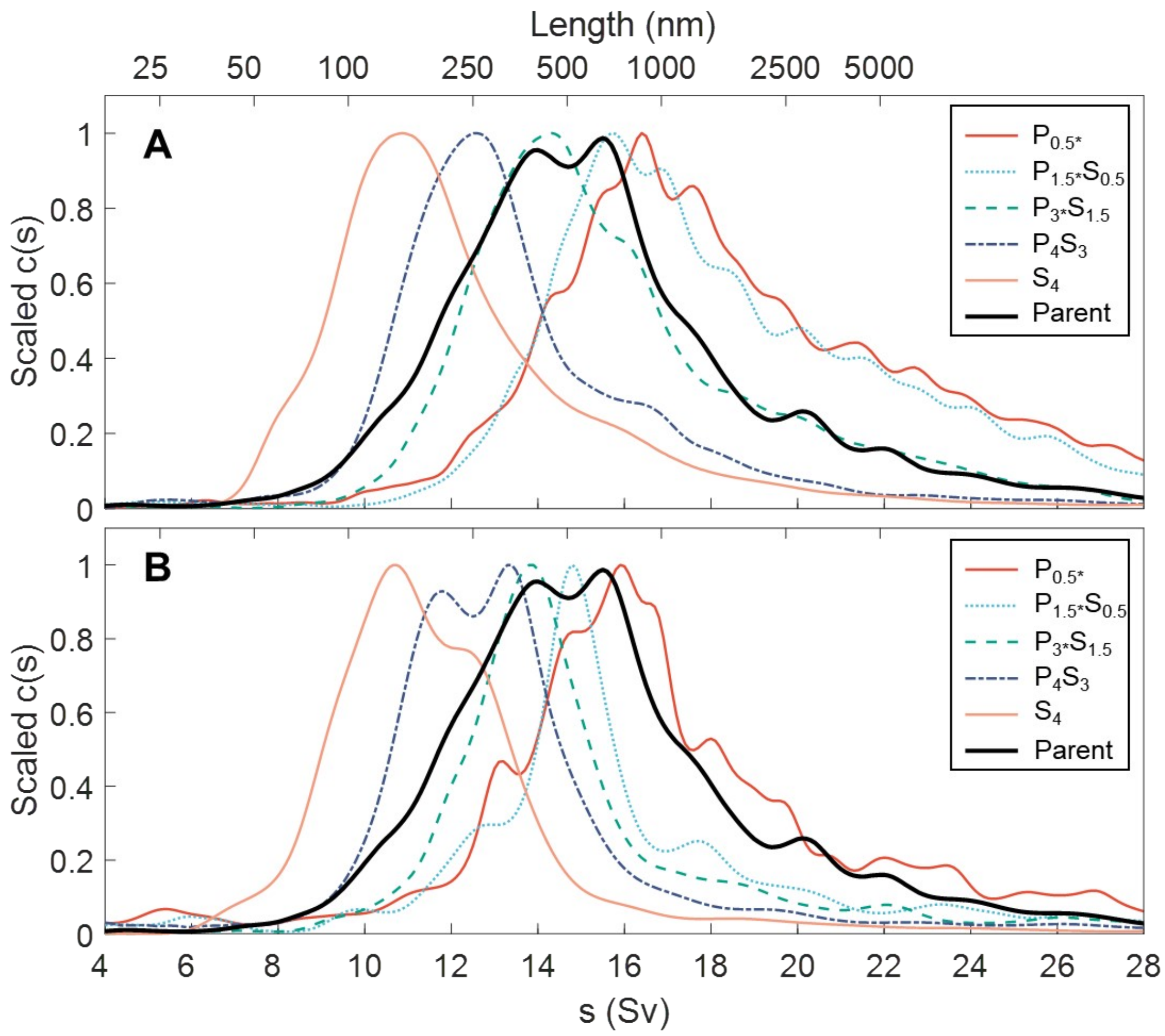

Fig. S8. FCVD SWCNT sedimentation behavior.

Sedimentation coefficient (***s***) profiles for tetracosane-filled, RZ- and ATPE-sorted metallic FCVD SWCNTs, fit from time-dependent radial absorbance profiles of each polymer-depleted length fraction measured *via* AUC. (A) ***s***-coefficient profiles of dispersions obtained after ultrafiltration (to remove the PMAA depletant) and resuspension in aqueous DOC solution. (B) AUC runs of the same fractions after 30 s of tip ultrasonication, showing dramatically reduced right tails in all fractions. Ultrasonication likely aided in disassociating bundled and loosely aggregated SWCNTs that were compacted during ultrafiltration.

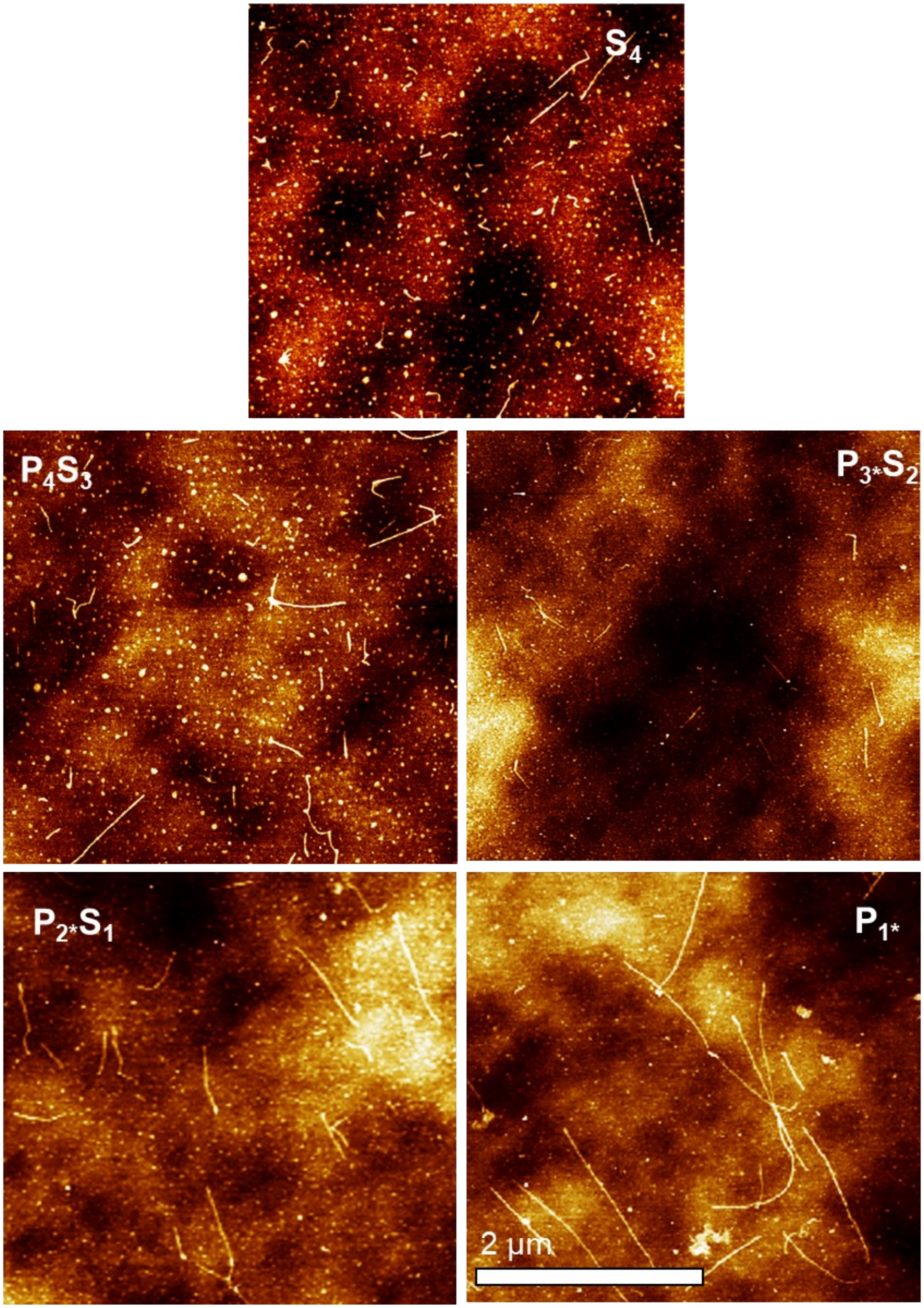

Fig. S9. Imaging of narrow-diameter SWCNT length separation.

Representative AFM images measured for each length fraction population sorted from the CoMoCAT SG SWCNTs. The scale bar is uniform for all images; circular-like points in S1 and S2 are assigned to be non-SWCNT objects, *e.g.*, artifacts formed from dried salt and surfactant.

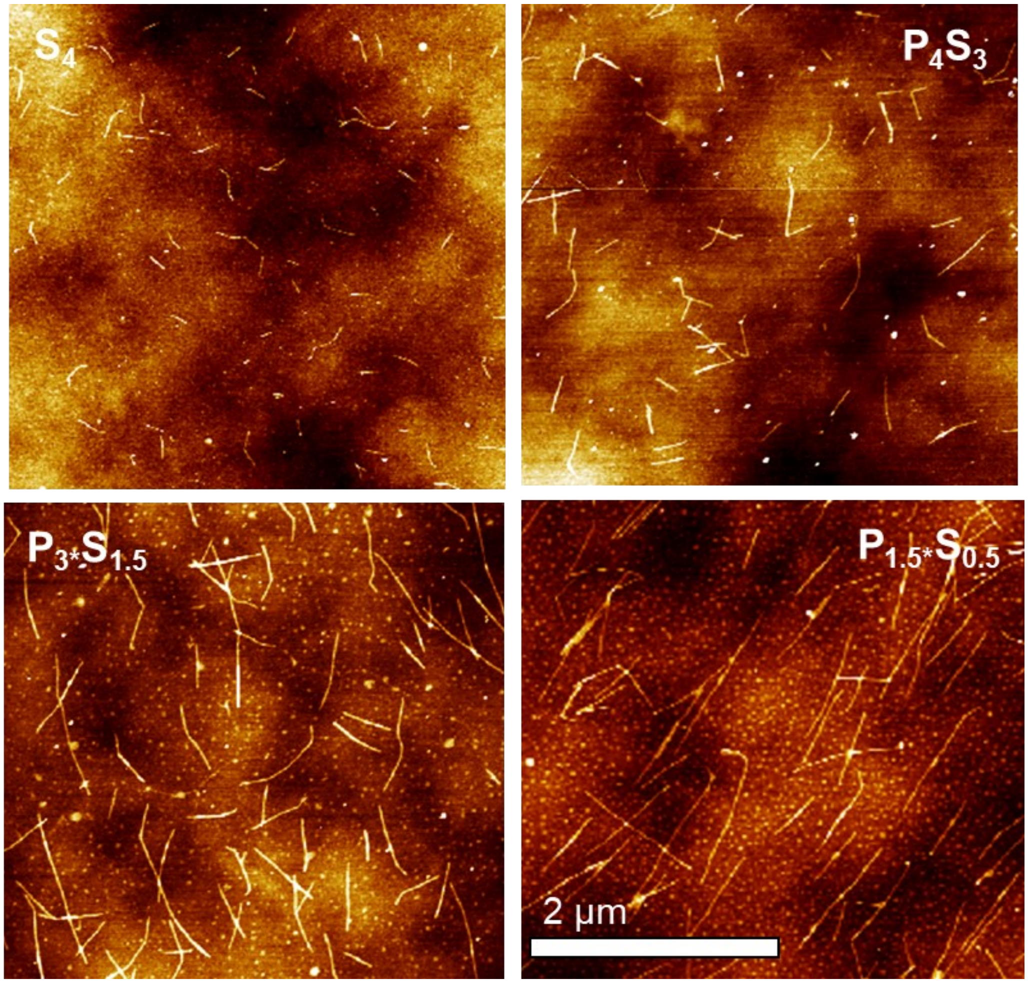

Fig. S10. Imaging intermediate-diameter SWCNT length separation.

Representative AFM images of S1 – S4 length fractions separated from the FCVD SWCNT parent dispersion. P4 was not scanned due to the negligible mass yield in that fraction.

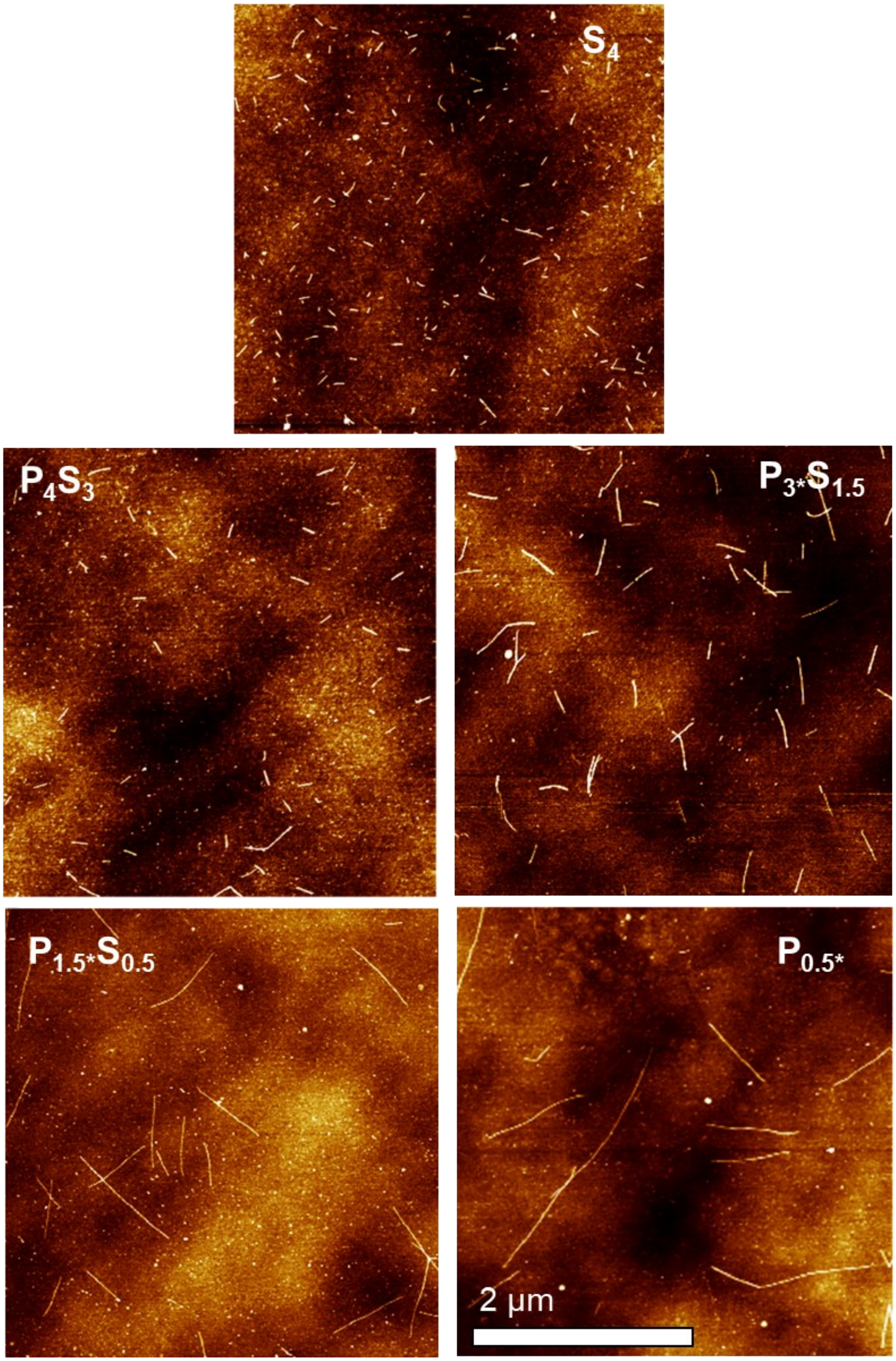

Fig. S11. Imaging large-diameter SWCNT length separation.
Representative AFM images of all length fractions sorted from EA SWCNTs.

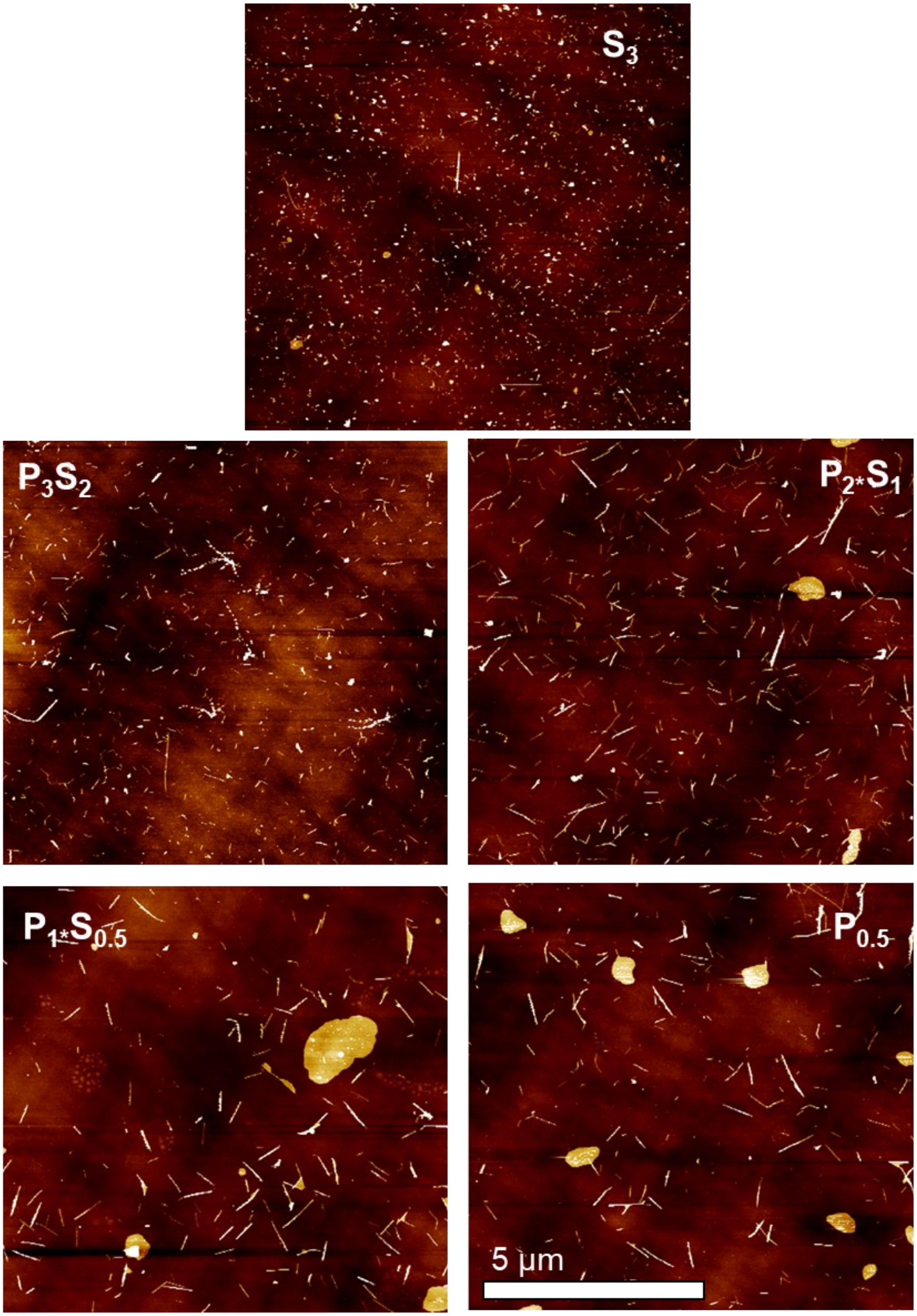

Fig. S12. Imaging BNNT length separation.

Representative AFM images of all length fractions sorted from BNNTs.

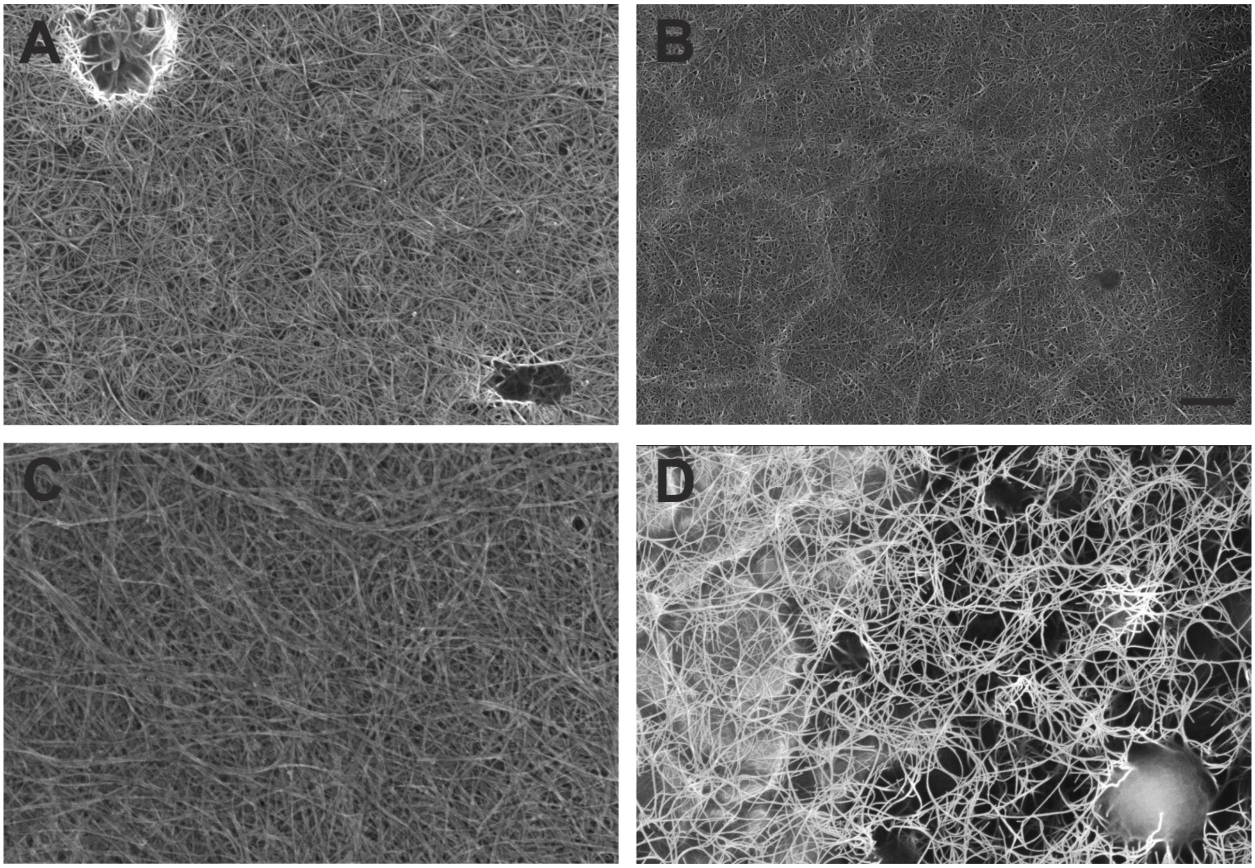

Fig. S13. SEM images of drop-casted nanotube samples.

SEM images of dropcast and rinsed aliquots of PDLS fractionated nanotube populations. While qualitatively demonstrating an exceptional morphological purity of the samples (low prevalence of non-nanotube material), these images are unsuitable for length determination due to the highly aggregated nature of the material under any viable sample preparation strategy. (A)PDLS-sorted EA SWCNTs, fraction $P_{1.5*}S_{0.5}$; (B) CoMoCAT/SG SWCNTs, fraction $P_{2*}S_1$; (C) BNNT fraction $P_{1*}S_{0.5}$; (D) FCVD SWCNTs, fraction $P_{1.5*}S_{0.5}$. Scale bar in (B) (applying to all panels) is 600 nm.

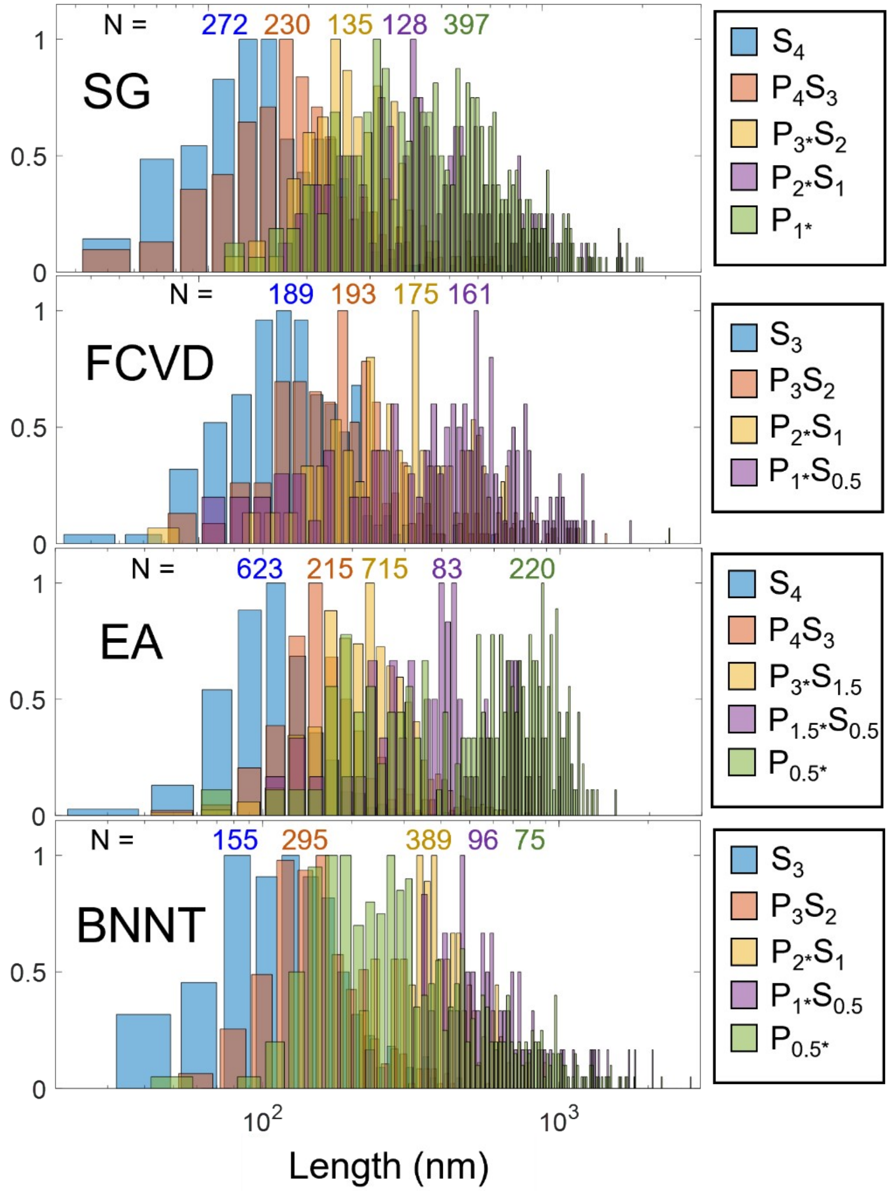

Fig. S14. Compilation of nanotube length and sample statistics.

AFM-derived length histograms and sample sizes for each separated nanotube fraction and source type.

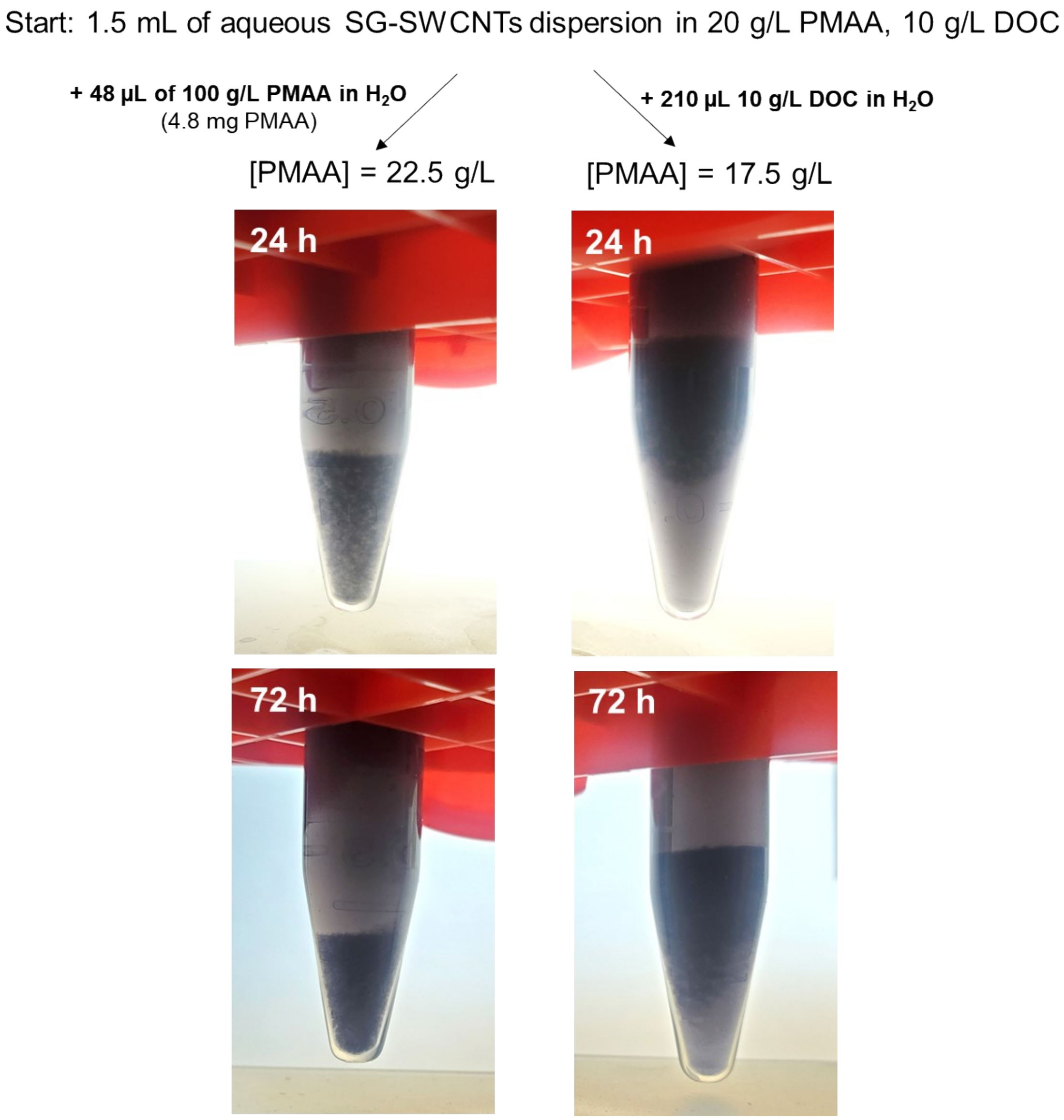

Fig. S15. Initiating and observing fine thermodynamic phase boundary shifts.

Images of SG SWCNT dispersion aliquots showing time-dependent appearance and pelleting of associated nanotube clusters after a fine modulation in PMAA polymer concentration from 20 g/L to 17.5 g/L or 22.5 g/L of PMAA, intended to further fractionate dispersions with a high degree of resolution. Notably, the parent dispersion in 20 g/L PMAA was entirely homogeneous and exhibited no precipitation in the weeks prior to the shown changes to the dispersion polymer concentration.

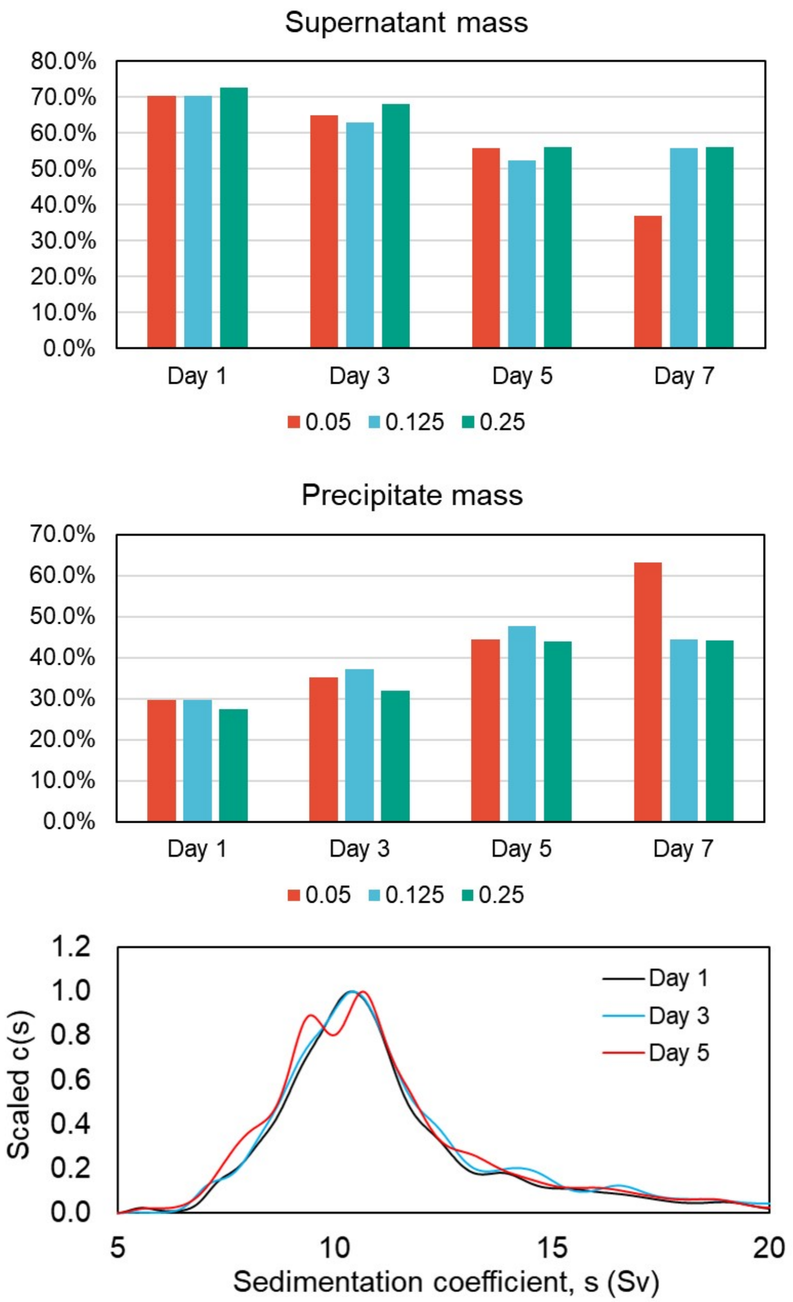

Fig. S16. Time-dependent mass and length distributions in PDLS sorted aliquots.

Separation yield (*i.e.*, mass distribution among supernatant and precipitate) as a function of initial nanotube concentration (in mg/mL) and settling time for CoMoCAT SG SWCNT nanotube material PDLS-separated with 20 g/L PMAA. Sedimentation profiles of the 0.125 mg/mL nanotube dispersion supernatant over five days show negligible differences in length distribution, indicating that the increases in mass yield are not kinetically limited and fully determined by the depletion effect.

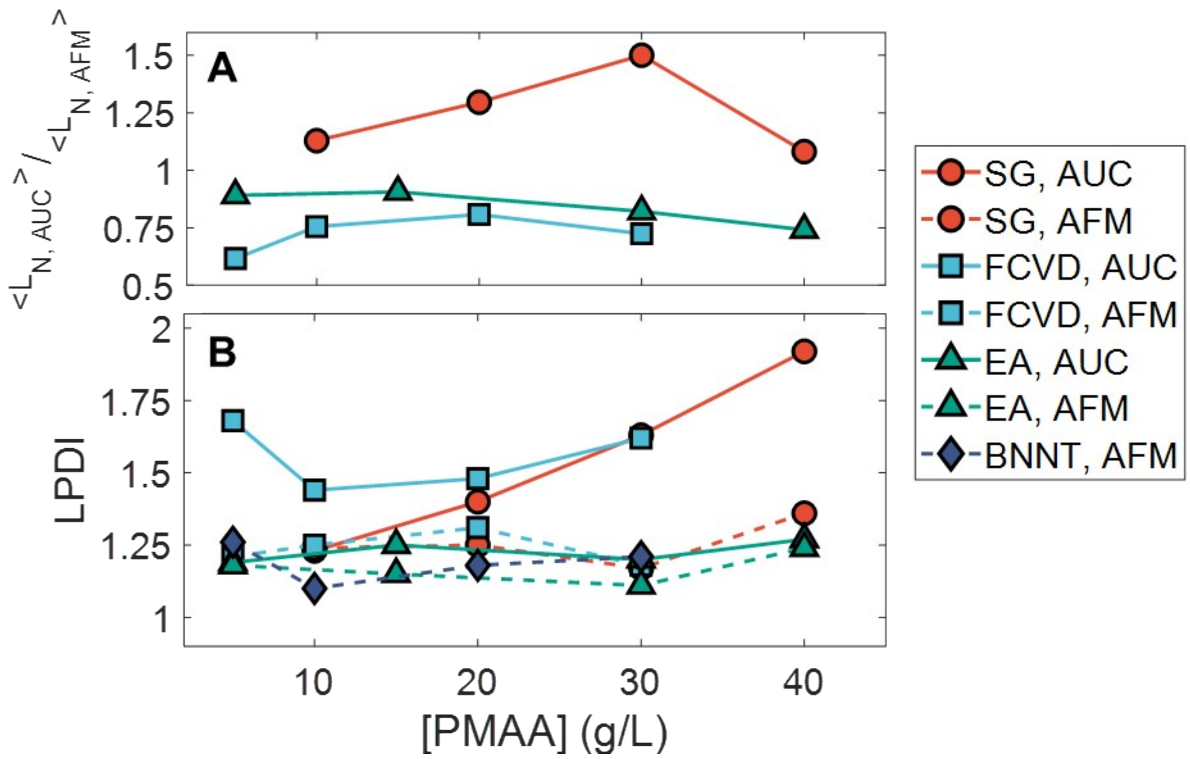

Fig. S17. Measurement and length variation among nanotube populations.

(A) Comparison of number-averaged SWCNT lengths calculated by AUC and AFM for each supernatant fraction. (B) Length polydispersity values for all nanotube fractions, contrasting the tightly distributed AFM-derived polydispersity values with those obtained via AUC.

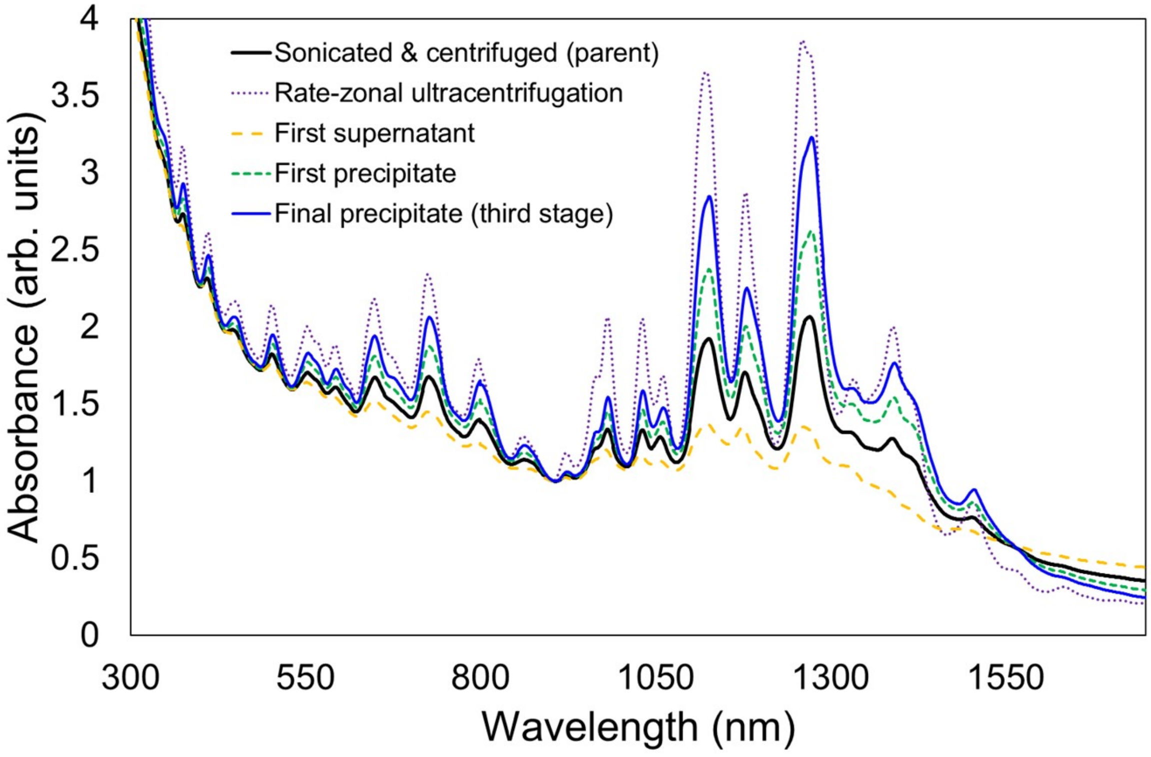

Fig. S18. PDLS as a purification method.

Scaled UV-Vis-NIR absorbance spectra of PDLS conducted on a high-pressure CO (HiPCO) synthesized SWCNT dispersion, showing the dramatic improvement in spectral peak-to-baseline which serves as a semi-quantitative proxy for dispersion impurity content. The precipitate obtained after stages of PDLS appears to approach the dispersion quality of that obtained by rate-zonal ultracentrifugation sorting, which requires use of costly density gradient media.

Table S1.

Concentrations of PMAA in g/L applied to each nanotube material type to generate PDLS fractionation at each separation stage.

**Nanotube material**	**EA**	**FCVD**	**SG**	**BNNT**
**Stage 1**	40.0	30.0	40.0	30.0
**Stage 2**	30.0	20.0	30.0	20.0
**Stage 3**	15.0	10.0	20.0	10.0
**Stage 4**	5.0	5.0	10.0	5.0

Table S2.

Estimated additional mass recovery (as reported by UV-vis-NIR absorbance) within each precipitation stage for three of the four nanotube source types investigated. Here, PX refers to the fractionation stage X (i.e., 1 through 4) as shown in Table S1, *not* the applied polymer concentration.

Total mass in each precipitate (mg)

	SG SWCNT	FCVD	BNNT
**P1**	18.3	0.49	10.5
**P1'**	0.13	0.19	1.27
**P2**	4.38	0.46	--
**P2'**	5.17	0.21	--
**P3**	2.46	0.072	4.73
**P3'**	1.72	0.075	1.04
**P4**	0.67	0.011	1.66
**P4'**	0.63	0.012	1.1

% additional recovery from secondary precipitate (PX') relative to primary (PX)

	SG SWCNT	FCVD	BNNT
**P1**	--	--	--
**P1'**	0.7 %	27.9 %	10.8 %
**P2**	--	--	--
**P2'**	54.1 %	31.3 %	--
**P3**	--	--	--
**P3'**	41.1 %	51.0 %	18.0 %
**P4**	--	--	--
**P4'**	48.5 %	52.2 %	39.9 %

Equation S1.

In order to target a particular fractionation point for nanotubes of length L [nm], hydrodynamic diameter d_{NT} [nm], polymer coil radius R_g [nm], coil volume V_{coil} [nm^3], and molar mass MM [Da], an estimate for the resulting concentration of polymer to apply to the solution, C [g/L], can be obtained by the following expression:

$$C = \frac{MM}{N_A V_{coil}} \left(\frac{2R_g d_{NT} L}{k_1} \right)^{-k_2}$$

Where $V_{coil} = \frac{4}{3}\pi R_g^3$, $N_A = 6.022 \times 10^{23}$ mol^{-1}, $k_1 = 565.4$, and $k_2 = -1.43$.

For the particular depletant used in the paper, the expression below holds:

$$C = \frac{6000}{8.5134} \left(\frac{2(1.504\ nm) d_{NT} L}{k_1} \right)^{-k_2}$$
